# Diblock Copolymer Nanoparticles Act as Nanoreactors for the Synthesis of Sterically‐Stabilized Inorganic Oxide Particles in Non‐polar Media

**DOI:** 10.1002/anie.4923622

**Published:** 2026-09-12

**Authors:** Priyanka Chohan, Andi Xie, Oleksandr O. Mykhaylyk, Steven P. Armes, Giles M. Prentice, David J. Atkinson, Andrew J. Smith

**Affiliations:** ^1^ School of Mathematical and Physical Sciences University of Sheffield Sheffield South Yorkshire UK; ^2^ Fluid Research, Castrol Technology Centre Reading UK; ^3^ Diamond Light Source, Diamond House, Harwell Science and Innovation Campus Oxford UK

**Keywords:** nanoreactor, polymerization‐induced self‐assembly, RAFT polymerization, silica, titania

## Abstract

Well‐defined diblock copolymer nanoparticles are prepared via reversible addition‐fragmentation chain transfer (RAFT) dispersion polymerization of 2‐hydroxyethyl methacrylate (HEMA) in a poly(α‐olefin) oil at 90°C using a poly(lauryl methacrylate) (PLMA) precursor. The PHEMA cores of these nanoparticles are subsequently swollen with up to 30% w/w aqueous 0.3 M HCl based on the PHEMA mass and then used as nanoreactors for the in situ synthesis of silica or titania using either tetraethyl orthosilicate (TEOS) or titanium *tert*‐butoxide, respectively. The PLMA‐PHEMA nanoparticles are characterized by transmission electron microscopy, dynamic light scattering (DLS), and small‐angle x‐ray scattering (SAXS). The former technique indicates that core‐swelling leads to a subtle change in copolymer morphology from pseudo‐spherical to perfectly spherical nanoparticles. DLS and SAXS confirm the formation of near‐monodisperse nanoparticles of 92–117 nm diameter with colloidal stability being retained after the nanoreactor syntheses. For the silicified nanoparticles, time‐resolved turbidimetry and SAXS studies suggest a reaction timescale of around 50 min at 25°C. In this case, the final nanoparticle dispersion is highly transparent, whereas the corresponding titania‐loaded nanoparticles produce a highly turbid dispersion. Thermogravimetric analyses indicate silica and titania mass loadings of 17% and 21%, respectively. In both cases the inorganic phase is amorphous rather than crystalline.

## Graphical abstract

Poly(lauryl methacrylate)‐poly(2‐hydroxyethyl methacrylate) (**PLMA**‐**PHEMA**) diblock copolymer nanoparticles prepared in non‐polar media serve as nanoreactors for the in situ synthesis of silica from tetraethyl orthosilicate (TEOS). A silica mass loading of 17% as judged by thermogravimetric analysis (TGA) produces a highly transparent dispersion, and dynamic light scattering (DLS) and small‐angle x‐ray scattering (SAXS) confirm no loss in colloidal stability.

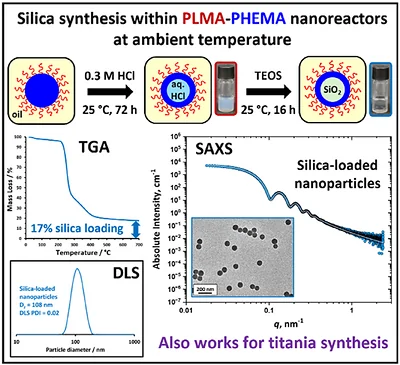

## 1 Introduction

Polymerization‐induced self‐assembly (PISA) is a well‐established technique for the synthesis of sterically‐stabilized diblock copolymer nanoparticles of controllable size and morphology [1, 2, 3, 4, 5, 6]. It involves growing a second block from a soluble precursor block: in a suitable solvent, the second block becomes insoluble at a certain critical degree of polymerization (DP), which induces micellar nucleation to form nascent spherical nanoparticles. Thereafter, the unreacted monomer enters the nanoparticle cores, which typically leads to a rate enhancement. The polymerization continues until all the remaining monomer is consumed: the final copolymer morphology can be spheres, worms, or vesicles depending on the reaction conditions. One important advantage of PISA over traditional post‐polymerization processing is that it enables diblock copolymer nanoparticles to be prepared at up to 50% *w*/*w* solids.

PISA syntheses can be conducted in water [4, 7, 8], lower alcohols [9, 10, 11, 12, 13], or non‐polar solvents (e.g., *n*‐alkanes, mineral oil, or poly(α‐olefins) [5, 14, 15, 16, 17, 18, 19, 20, 21]. Various applications have been explored for the latter formulations, including cosmetics applications (nail varnishes) [22, 23, 24], nanoparticle lubricants [20, 25, 26, 27], wax crystal inhibitors [28], and bespoke nanoparticles for the preparation of water‐in‐oil Pickering nanoemulsions [29]. Recently, we reported a new PISA formulation in non‐polar media [30]. A poly(lauryl methacrylate) (PLMA) precursor was chain‐extended via reversible addition‐fragmentation chain transfer (RAFT) dispersion polymerization of 2‐hydroxyethyl methacrylate (HEMA) in *n*‐dodecane at 90°C to produce PLMA‐PHEMA nanoparticles. For a relatively short PLMA precursor with a DP of 14, the mean nanoparticle diameter could be tuned from 25 to 131 nm by varying the target DP for the core‐forming PHEMA block. The polar nature of the HEMA monomer led to a relatively fast rate of polymerization [31], which ensured a very high final HEMA conversion (≥ 98%). However, bimodal particle size distributions were obtained when targeting higher PHEMA DPs using a longer PLMA precursor owing to a side‐reaction between the RAFT chain‐ends and the HEMA monomer [30]. Fortunately, this problem could be circumvented by employing a monomer‐starved feed protocol [30]. Targeting higher PHEMA DPs also led to microgel‐like, lightly cross‐linked nanoparticle cores owing to a dimethacrylate impurity in the HEMA monomer [30].

There have been numerous reports of the use of copolymer nanoparticles to prepare organic–inorganic nanocomposites [32, 33, 34, 35, 36, 37, 38, 39, 40, 41, 42, 43, 44, 45, 46, 47]. For example, Lecommandoux and coworkers incorporated hydrophobic maghemite sols within block copolymer nano‐objects to produce magnetically‐responsive vesicles or spheres [35]. Similarly, Hennink and coworkers reported the synthesis of biodegradable block copolymer micelles loaded with magnetite nanoparticles [41]. Alternatively, spherical nanoparticles have been used as colloidal templates for the controlled deposition of silica using either tetraethyl orthosilicate (TEOS) or tetramethyl orthosilicate (TMOS) as a soluble precursor [36, 38, 39]. Müller and coworkers have grown silica or titania within anisotropic cylindrical brush nanoparticles [44, 45], while Wang et al. coated cylindrical diblock or triblock copolymer micelles with silica, alumina, or zirconia [42]. However, all such reports typically involve syntheses conducted in either aqueous media or an aqueous alcoholic milieu. As far as we are aware, there are far fewer—if any—reports involving the use of block copolymer nanoparticles as nanoreactors in non‐polar media. Moreover, most prior studies involve laborious multistep copolymer syntheses (e.g., use of expensive comonomers and/or protecting group chemistry) plus post‐polymerization processing in dilute solution [32, 33, 34, 35, 36, 37, 38, 39, 40, 41, 42, 43, 44, 45, 46, 47].

Herein, we use PLMA‐PHEMA nanoparticles as putative nanoreactors for the in situ synthesis of sterically‐stabilized inorganic oxide particles in non‐polar media, more specifically a poly(α‐olefin) (PAO) oil. In this case, the PHEMA cores are first swollen with a solution of aqueous acid and then the oil‐soluble inorganic precursor is introduced into the continuous phase (see Scheme 1). Hydrolysis of this precursor is confined to the nanoparticle cores, which should ensure retention of colloidal stability for syntheses performed under optimized conditions. This new approach enables the efficient and convenient synthesis of nanocomposite particles at ∼12% *w*/*w* solids, which is significantly higher than that reported in the literature (typically less than 1% *w*/*w* solids) [32, 33, 34, 35, 36, 37, 38, 39, 40, 41, 42, 43, 44, 45, 46, 47]. In principle, such nanocomposite particles could be used to produce transparent colloidal dispersions [48, 49], as photocatalysts [50, 51], for electrophoretic displays [52], or next‐generation thermal management fluids for computer electronics or batteries [53, 54, 55, 56].

**SCHEME 1:**
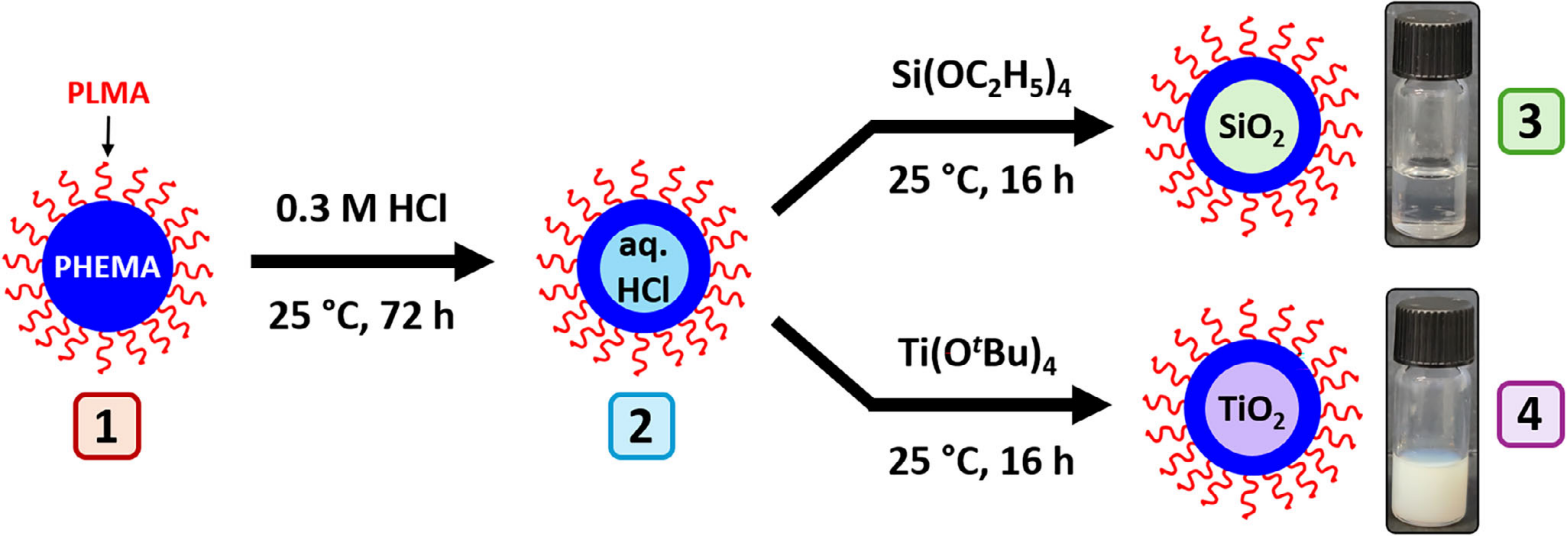
Schematic cartoon illustrating the use of nanoparticles as nanoreactors: PLMA_194_‐PHEMA_2000_ nanoparticles (1) are swollen with an aqueous solution of 0.3 M HCl to form acid‐swollen nanoparticles (2) and then reacted with either tetraethyl orthosilicate (TEOS) to form silica‐loaded nanoparticles (3) or reacted with titanium *tert*‐butoxide to form titania‐loaded nanoparticles (4). Representative digital photographs of the physical appearance of the silica‐loaded nanoparticles (transparent dispersion) and titania‐loaded nanoparticles (turbid dispersion) are also shown.

## 2 Results and Discussion

### 2.1 Synthesis of PLMA_194_‐PHEMA*_x_* Nanoparticles at 10% *w*/*w* Solids Under Monomer‐Starved Conditions

A series of PLMA_194_‐PHEMA*_x_* nanoparticles were prepared according to the synthesis protocol summarized in Scheme 2 using the monomer‐starved feed protocol reported previously [30]. First, a PLMA_194_ precursor was synthesized by RAFT solution polymerization of LMA at 80% *w*/*w* solids in PAO using cumyl dithiobenzoate (CDB). Without any purification, this precursor was chain‐extended via RAFT dispersion polymerization of HEMA at 10% *w*/*w* solids to target a series of PLMA_194_‐PHEMA*_x_* nanoparticles (where *x* = 980–2910). HEMA monomer was added using an automated syringe pump to ensure the formation of well‐defined nanoparticles with unimodal particle size distributions [30]. ^1^H NMR spectroscopy analysis of the final diblock copolymers in *d*_5_‐pyridine indicated at least 97% HEMA conversion in all cases. Unfortunately, the lightly cross‐linked nature of the PHEMA cores precluded GPC analysis. However, our prior study suggests that a relatively high chain extension efficiency can be achieved for such PISA formulations [30].

**SCHEME 2:**
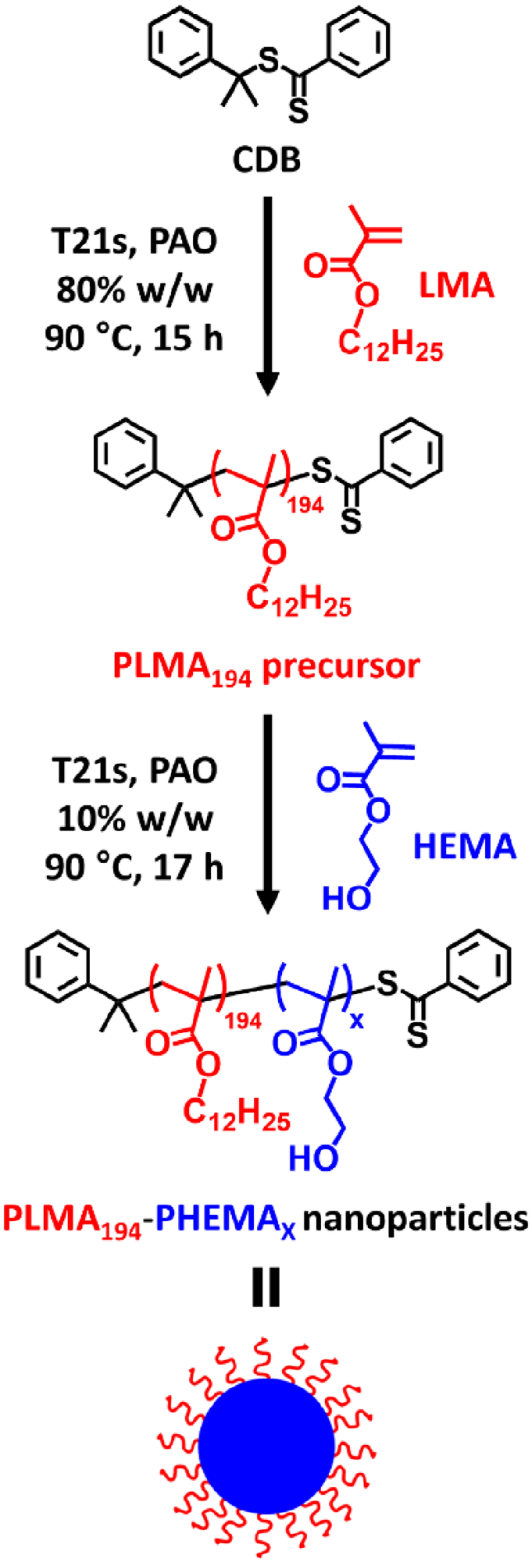
One‐pot synthesis of a poly(lauryl methacrylate) (PLMA) precursor via RAFT solution polymerization of LMA in PAO at 90°C using cumyl dithiobenzoate (CDB) and T21s initiator ([CDB]/[T21s] molar ratio = 3.0), immediately followed by the RAFT dispersion polymerization of 2‐hydroxyethyl methacrylate (HEMA) under monomer‐starved conditions in PAO at 90°C ([precursor]/[T21s] molar ratio = 3.0) to form a series of PLMA_194_‐PHEMA*_x_* nanoparticles (where *x* = 980–2910).

Dynamic light scattering (DLS) studies indicated relatively narrow, unimodal particle size distributions for the PLMA_194_‐PHEMA_980–2910_ series, with a corresponding increase in the z‐average particle diameter when targeting a higher PHEMA DP (see Figure 1a). This is corroborated by TEM analysis of the nanoparticles (see Figure 1b). The imperfect pseudo‐spherical morphology exhibited by these nanoparticles is likely related to the extensive hydrogen bonding between the primary hydroxy groups and the methacrylic ester groups within the PHEMA core (see later). The data obtained from DLS and TEM are summarized in Table S1. Small‐angle x‐ray scattering (SAXS) analysis was also performed on this series of nanoparticles, and the corresponding *I*(*q*) versus *q* plots are shown in Figure 2a. Each scattering pattern can be satisfactorily fitted using a well‐known spherical micelle model [57] (see Equations S14 and S15). Multiple minima are observed in all cases, which indicates the formation of well‐defined, near‐monodisperse nanoparticles. The primary minimum in each SAXS pattern corresponds approximately to the volume‐average core radius, *R*, according to the relation *R* = 4.49/*q* [58]. As the PHEMA DP increases, these primary minima shift toward lower *q*, indicating an increase in the volume‐average core diameter from 59 to 81 nm (as shown in Figure 2b and Table S2).

**FIGURE 1:**
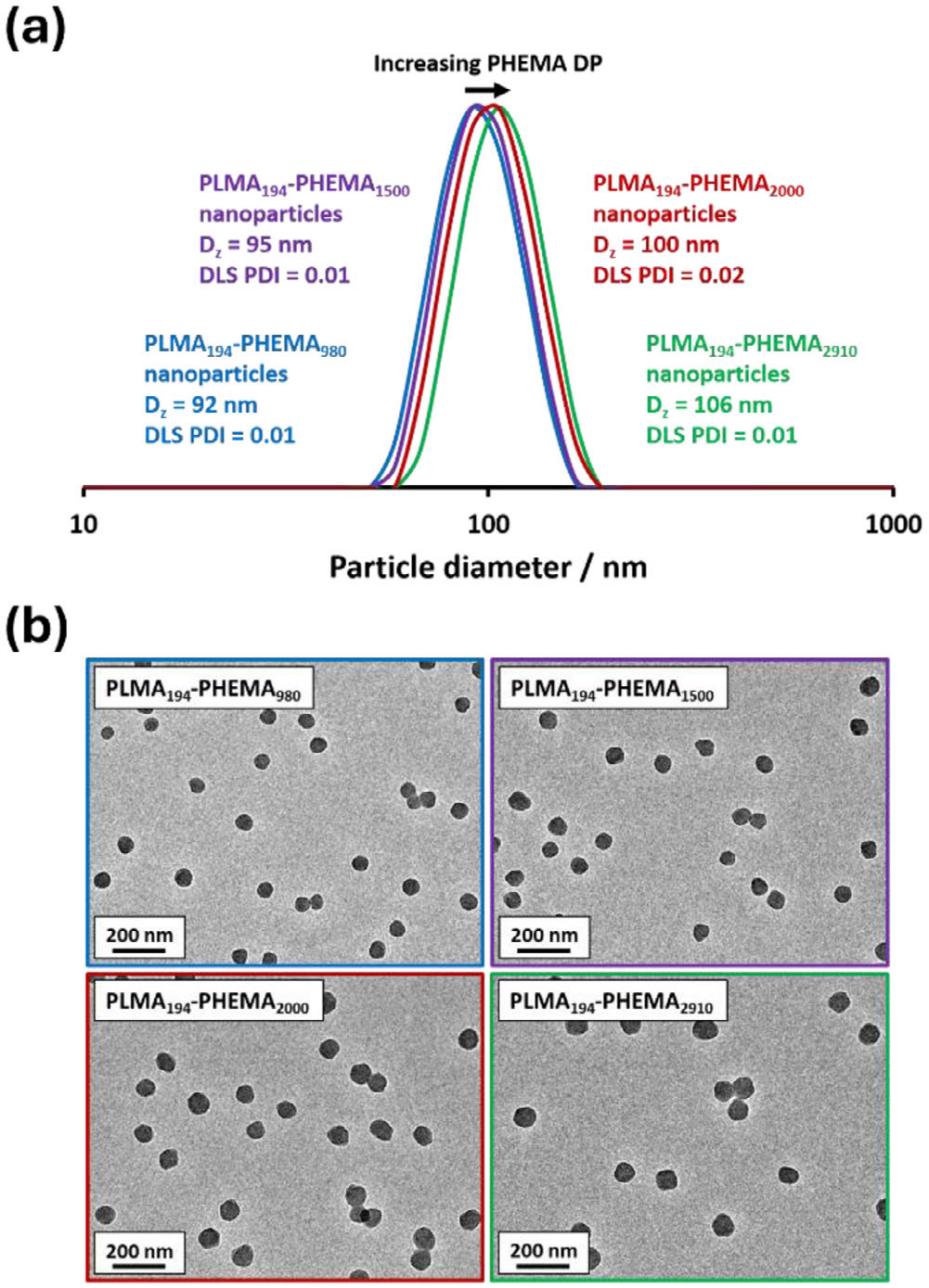
(a) DLS particle size distributions recorded for 0.1% *w*/*w* dispersions of a series of PLMA_194_‐PHEMA_980–2910_ nanoparticles prepared by RAFT dispersion polymerization of HEMA under monomer‐starved conditions in PAO at 90°C when targeting 10% *w*/*w* solids (*D*_z_ is the z‐average hydrodynamic diameter and PDI denotes the DLS polydispersity). (b) Representative TEM images recorded for the same series of four nanoparticle dispersions.

**FIGURE 2:**
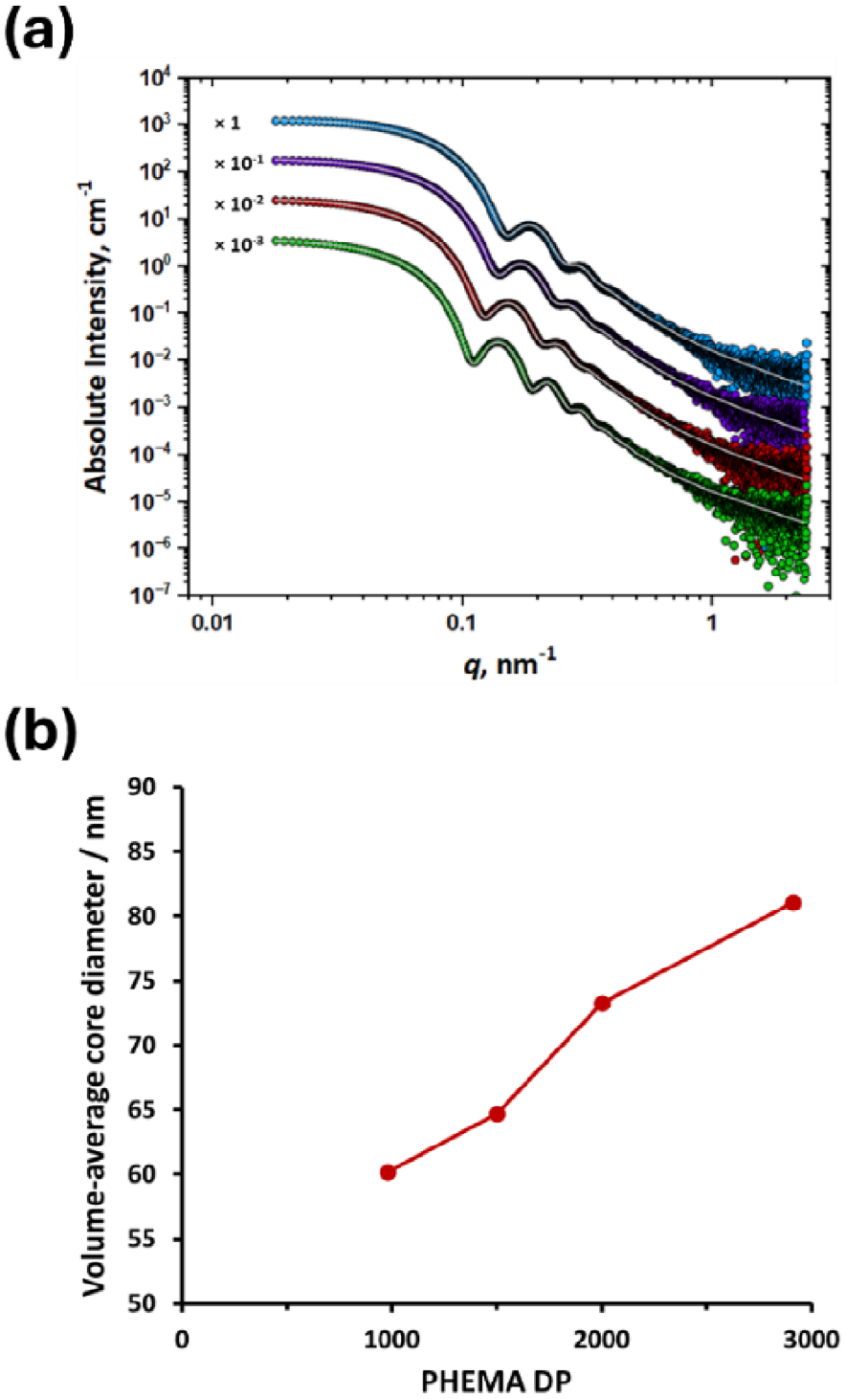
(a) SAXS patterns recorded for 1.0% *w*/*w* diblock copolymer dispersions prepared in PAO (PLMA_194_‐PHEMA_980_, blue curve; PLMA_194_‐PHEMA_1500_, purple curve; PLMA_194_‐PHEMA_2000_, red curve; and PLMA_194_‐PHEMA_2910_, green curve). Data fits (grey curves) to SAXS patterns were obtained using a well‐known spherical micelle model [57]. These SAXS patterns have been offset by an arbitrary multiplication factor for clarity. (b) Variation in volume‐average core diameter with PHEMA DP for the same four examples of PLMA_194_–PHEMA_980–2910_ nanoparticles.

However, there was no apparent increase in the mean aggregation number (Table S3). The corresponding area occupied by each PLMA_194_ chain at the nanoparticle surface (see *S*_block_(*R_s_*) in Table S3) increases from 423 Å^2^ for PLMA_194_‐PHEMA_980_ to 907 Å^2^ for PLMA_194_‐PHEMA_2910_. This is consistent with a concomitant reduction in the corona thickness, *s* (Table S3). Indeed, as the surface packing density of these coronal chains is reduced, they gain greater conformational freedom and tend to reside closer to the core, which results in a thinner coronal layer. Targeting higher PHEMA DPs leads to a patchy coronal layer and hence less effective steric stabilization [59]. This explains why a PHEMA DP of around 3000 represents the effective upper limit for such PISA formulations, at least when utilizing a PLMA_194_ precursor. Moreover, targeting PHEMA DPs above 3000 leads to substantially incomplete HEMA conversions owing to the relatively low radical flux generated under such conditions.

### 2.2 Swelling of PLMA_194_‐PHEMA_2000_ Nanoparticles With Aqueous HCl

According to the literature [60, 61, 62], high molecular weight PHEMA is a water‐swellable polymer that can absorb water up to 30% of its mass; this is a useful property in the context of the present study. Moreover, its hydroxyl functionality means that in situ generation of the inorganic oxide phase (e.g., silica or titania) should also lead to covalent stabilization of the nanoparticle cores. Such inorganic oxides can be prepared using a sol–gel method, whereby a suitable soluble precursor is first hydrolyzed before undergoing condensation [63, 64, 65, 66, 67, 68]. Often an acid or base is added to catalyze the sol–gel reaction. In this study, PLMA_194_‐PHEMA_2000_ nanoparticles were swollen by 30% *w*/*w* (based on the mass of their PHEMA cores) using an aqueous solution of 0.3 M HCl. Initially, this acidic solution is clearly visible as a separate phase within the nanoparticle dispersion, but after stirring for 3 days, a homogenous dispersion is obtained. DLS analysis of the swollen nanoparticles indicates a modest increase in the z‐average diameter in comparison to the original PLMA_194_‐PHEMA_2000_ nanoparticles, along with minimal change in the DLS polydispersity (see Figure 3). This suggests that the swollen nanoparticles retain their colloidal stability. Interestingly, TEM analysis of the acid‐swollen nanoparticles indicated perfectly spherical nanoparticles (see Figure 3). This subtle change in morphology compared to the original pseudo‐spherical nanoparticles suggests that swelling the nanoparticle cores allows relaxation of the initial hydrogen bonding interactions formed in the absence of any water and hence modifies the morphology. Furthermore, when the PHEMA cores (refractive index, RI = 1.51) [69] become swollen with aqueous acid (RI = 1.34) [70], the RI difference between the nanoparticles and the continuous phase (PAO RI = 1.45) is reduced, which results in a less turbid dispersion (see Figure 3).

**FIGURE 3:**
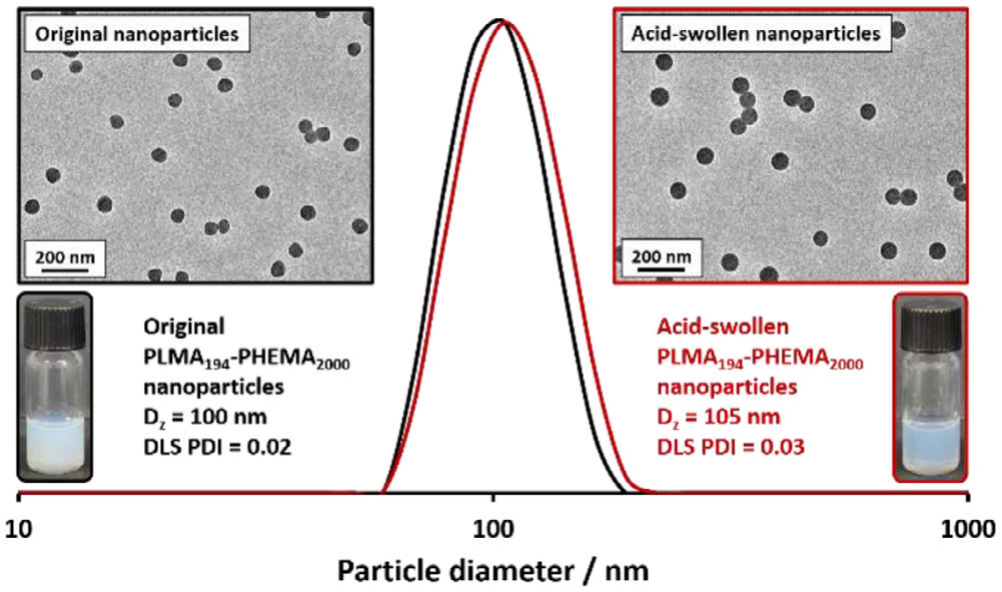
DLS particle size distributions and corresponding TEM images recorded for 0.1% *w*/*w* dispersions of the original PLMA_194_‐PHEMA_2000_ nanoparticles (black) and aqueous acid‐swollen PLMA_194_‐PHEMA_2000_ nanoparticles (red). A digital photograph showing the physical appearance of each 10% *w*/*w* nanoparticle dispersion is also shown.

In addition, SAXS analysis of the acid‐swollen nanoparticles shows a shift in the primary minimum to lower *q* compared to that for the original nanoparticles, indicating an increase in their core diameter from 73.0 to 79.8 nm (Figure 4 and Table S3). The SAXS pattern obtained for the acid‐swollen nanoparticles also exhibits additional minima, suggesting the formation of even more well‐defined nanoparticles. Given that the nanoparticle cores are lightly cross‐linked, the mean aggregation number determined by SAXS analysis of the original, non‐swollen PLMA_194_‐PHEMA_2000_ nanoparticles was held constant (along with the corona block thickness) when fitting SAXS patterns recorded for the acid‐swollen, silica‐loaded and titania‐loaded PLMA_194_‐PHEMA_2000_ nanoparticles. For the acid‐swollen nanoparticles, this approach indicated a volume fraction, *x*_HCl_, of 0.24 for the aqueous acid within the nanoparticle cores when using Equations S14 and S15. This is in reasonable agreement with the targeted volume fraction of 0.28, which corresponds to 30% *w*/*w* aqueous acid based on the PHEMA mass. This indicates that all of the aqueous 0.3 M HCl added to the dispersion diffuses into the nanoparticle cores. The SAXS pattern recorded for the silicified nanoparticles will be discussed later.

**FIGURE 4:**
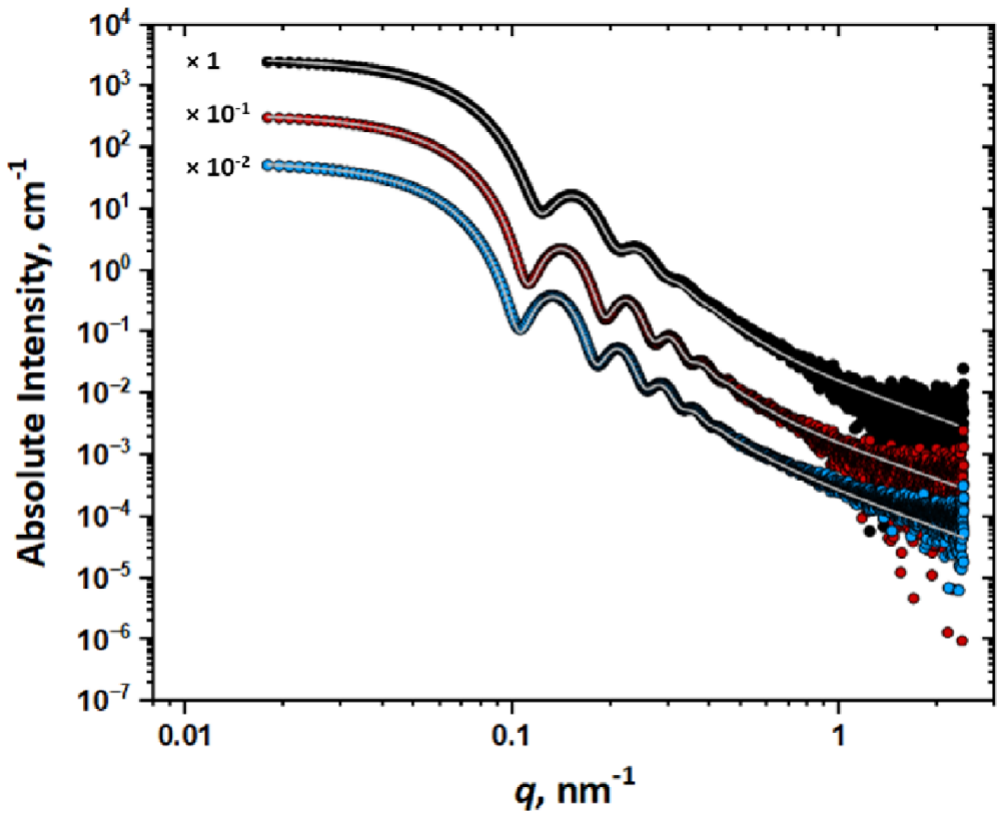
SAXS patterns recorded for 1.0% *w*/*w* dispersions of the original PLMA_194_‐PHEMA_2000_ nanoparticles (black curve), the acid‐swollen PLMA_194_‐PHEMA_2000_ nanoparticles (red curve), and the silicified nanoparticles (blue curve) in PAO. Data fits (grey curves) to the former two patterns were obtained using a well‐known spherical micelle model [57] and the latter fit for the silicified nanoparticles was obtained using a modified version of the blob model [71]. See the Supporting Information for details of the SAXS models. The SAXS patterns have been offset by an arbitary multiplication factor for clarity.

To confirm swelling of the PHEMA cores, a ^1^H NMR spectroscopy experiment was conducted (see Figure 5). As expected, the ^1^H NMR spectrum of the original PLMA_194_‐PHEMA_2000_ nanoparticles redispersed in *d*_26_‐dodecane contained a broad signal at 3.9–4.2 ppm assigned to the PLMA steric stabilizer block (two oxymethylene protons adjacent to the methacrylic ester) but no detectable signals corresponding to the non‐solvated PHEMA block. However, after swelling such nanoparticles with 0.3 M DCl/D_2_O, an –OCH_2_C*H*_2_OH signal assigned to the PHEMA block becomes visible at 3.8 ppm (N.B. the corresponding–OC*H*_2_CH_2_OH signal is most likely hidden under the PLMA signal). This provides direct spectroscopic evidence that the PHEMA cores become swollen with the aqueous acid, which is consistent with the DLS, TEM, and SAXS evidence presented above.

**FIGURE 5:**
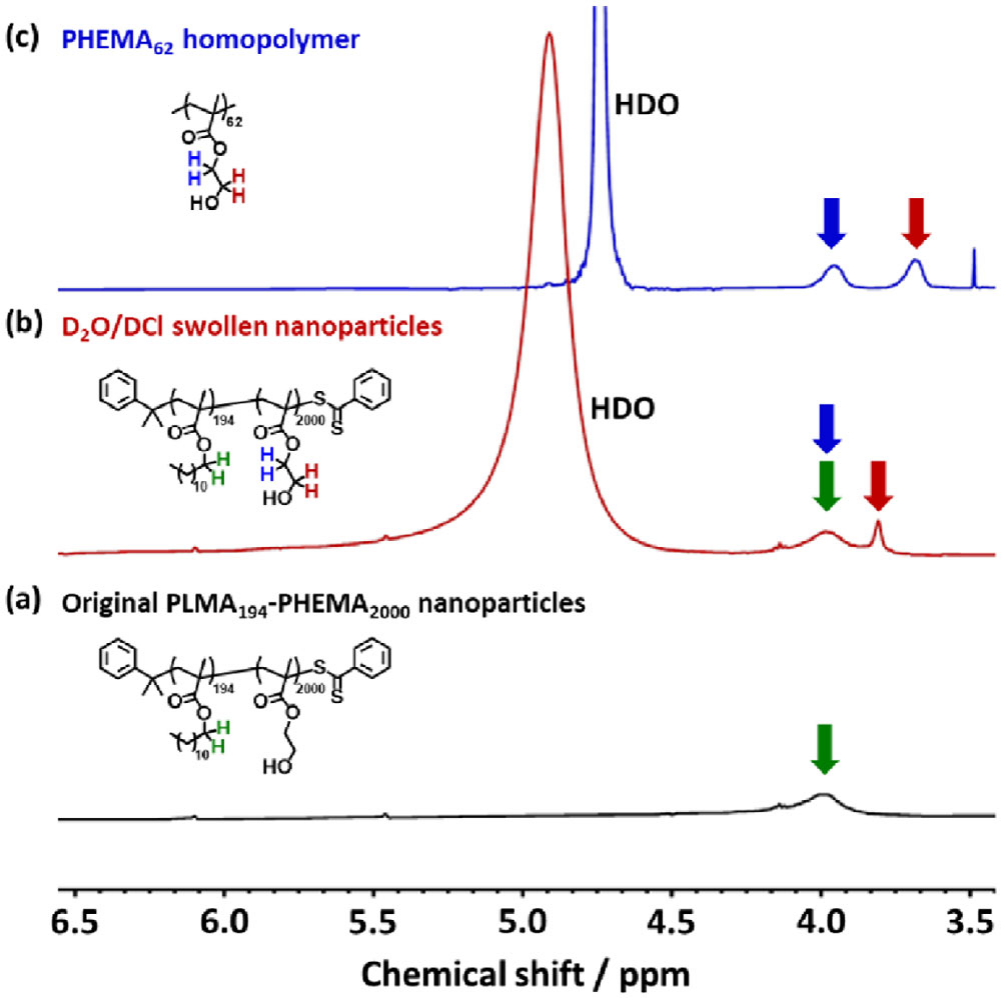
^1^H NMR spectra recorded for (a) the original (non‐swollen) PLMA_194_‐PHEMA_2000_ nanoparticles in *d*_26_‐dodecane (black spectrum), (b) the D_2_/DCl Oswollen nanoparticles in *d*_26_‐dodecane (red spectrum), and (c) a PHEMA_62_ homopolymer in D_2_O/DCl for reference (blue spectrum). The 3.8 ppm signal in spectrum (b) is assigned to the two–OCH_2_C*H*_2_OH protons of the PHEMA chains located within the swollen nanoparticle cores. The corresponding –OC*H*_2_CH_2_OH proton signal most likely overlaps with the PLMA oxymethylene proton signal at 3.9–4.2 ppm.

### 2.3 Preparation of Silica‐Loaded PLMA_194_‐PHEMA_2000_ Nanoparticles

Preparation of silica via sol–gel chemistry has been widely reported in the literature [65, 72, 73, 74, 75] and can be conducted at ambient temperature [76, 77, 78]. In this study, TEOS was selected as an oil‐soluble silica precursor that can diffuse through the PAO continuous phase and enter the acid‐swollen nanoparticle cores. Once inside, TEOS undergoes acid‐catalyzed hydrolysis to generate ethanol and Si(OH)_4_, which subsequently undergoes self‐condensation to form Si–O–Si linkages, leading to in situ formation of silica. However, the nanoparticle cores also contain one primary hydroxyl group per HEMA repeat unit. These hydroxyl groups almost certainly participate in the sol–gel chemistry, thus forming many covalent bonds between the inorganic and organic components to produce highly cross‐linked nanoparticle cores.

Such silicification leads to a discernible increase in the mean nanoparticle diameter (see Figure 6a). However, the DLS polydispersity remains relatively low, which indicates that colloidal stability is retained and suggests that silica formation is confined to the nanoparticle cores. A representative TEM image recorded for the silicified nanoparticles is shown in Figure 6b. SAXS analysis using a modified scattering model (see Equations S22 and S23) also produced a satisfactory data fit to the SAXS pattern recorded for the silica‐loaded PLMA_194_‐PHEMA_2000_ nanoparticles (see Figure 4, Figures S3,S4 and Table S3), indicating a core diameter of 85.2 nm. A silica volume fraction of 0.37 was calculated for the nanoparticle cores (and a silica volume fraction of around 0.32 for the overall nanoparticle). This is in reasonable agreement with a silica volume fraction of 0.369 calculated from the corresponding solution densitometry measurements for such nanoparticle dispersions. SAXS analysis also indicates that the radius of gyration, *R*_gin_, for the silica blobs within the core is ∼ 35 Å.

**FIGURE 6:**
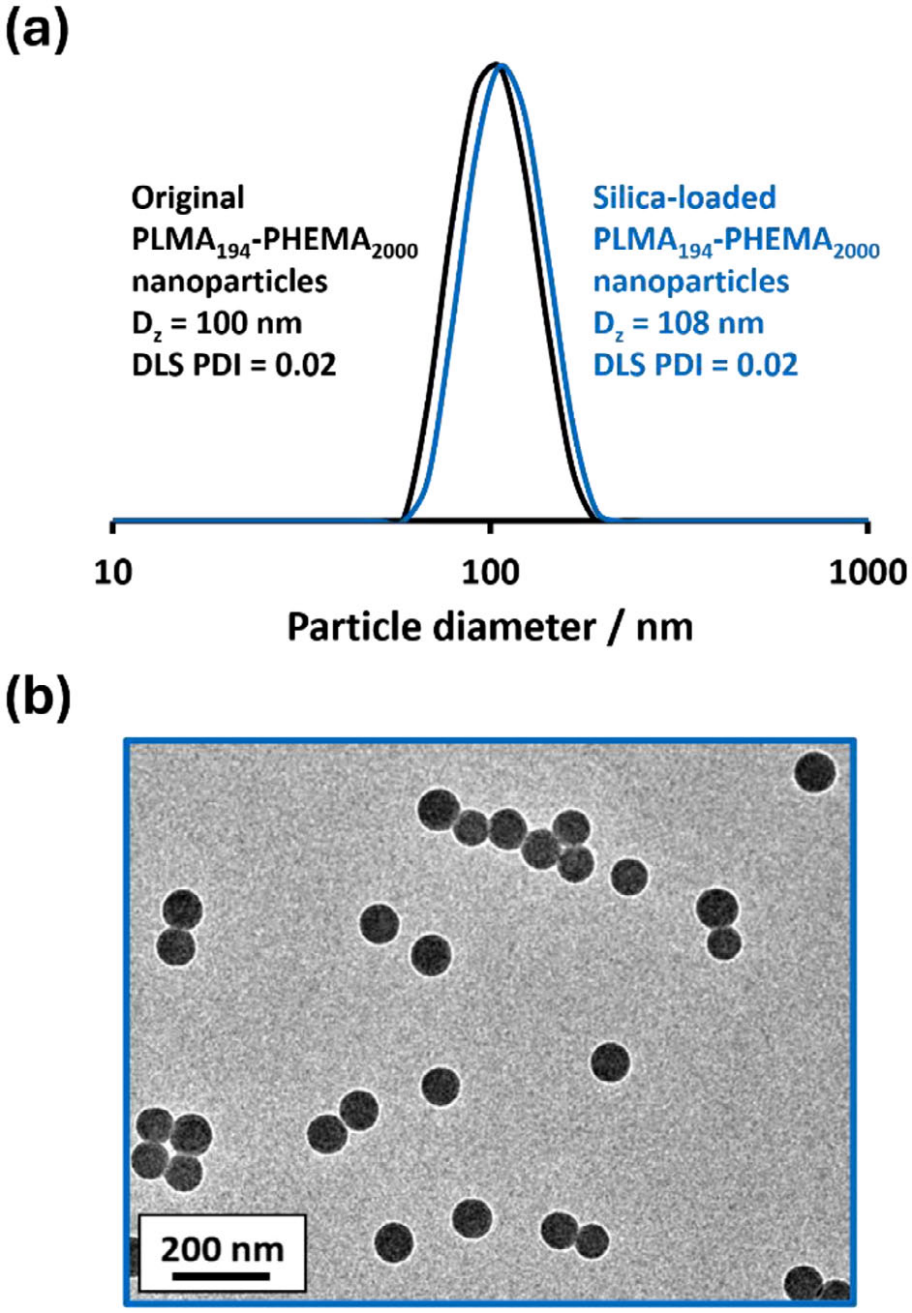
(a) DLS particle size distributions recorded for 0.1% *w*/*w* dispersions of the original nanoparticles and the corresponding silica‐loaded nanoparticles. (b) Representative TEM image recorded for the silica‐loaded nanoparticles.

To determine the silica loading, thermogravimetric analysis (TGA) was conducted on the dried silica‐loaded nanoparticles (Figure 7). The residual mass of 17.0% corresponds to the incombustible silica component, which indicates a yield of 98% based on the amount of added TEOS. As a control, TGA analysis of the dried PLMA_194_‐PHEMA_2000_ nanoparticles was also conducted to confirm that this methacrylic copolymer underwent complete thermal degradation with no detectable residue. In addition, Fourier transform infrared (FT‐IR) spectroscopy was employed to analyze the original dried PLMA_194_‐PHEMA_2000_ nanoparticles, silica‐loaded nanoparticles, and the silica residue obtained after TGA (Figure 8). As highlighted by the light grey region, formation of the silica‐loaded nanoparticles produces a more intense absorption band at approximately 1100 cm^−^^1^, which corresponds to the Si–O–Si stretch [79, 80, 81]. FT‐IR spectroscopy analysis of the TGA residue confirmed the complete absence of any characteristic copolymer bands (e.g., no strong carbonyl ester band at 1720 cm^−1^), indicating complete pyrolysis of this organic component.

**FIGURE 7:**
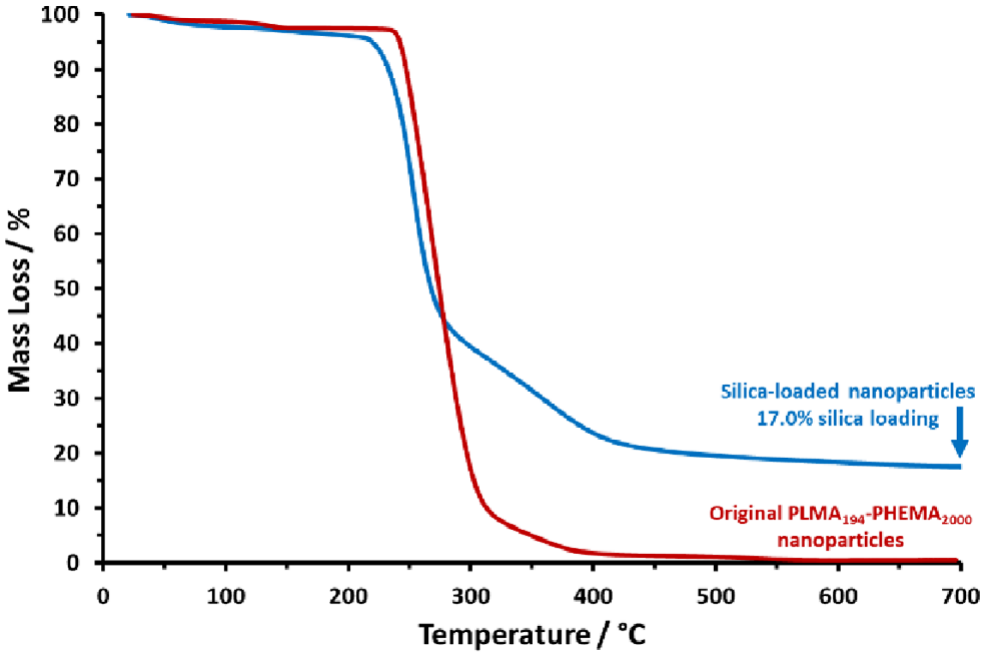
TGA curves recorded under air for the original PLMA_194_‐PHEMA_2000_ nanoparticles (red) and the silica‐loaded PLMA_194_‐PHEMA_2000_ nanoparticles (blue).

**FIGURE 8:**
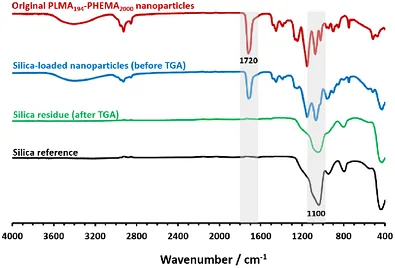
FT‐IR spectra recorded for the original PLMA_194_‐PHEMA_2000_ nanoparticles (red), silica‐loaded nanoparticles (blue), silica residue obtained after TGA analysis (green), and a silica reference (black).

### 2.4 Kinetic Studies of the In Situ Silicification of PLMA_194_‐PHEMA_2000_ Nanoparticles at 25°C

Owing to the highly transparent nature of the final silica‐loaded nanoparticle dispersion, the characteristic time scale required for silicification can be examined using visible absorption spectroscopy (see Figure S1). As shown in Figure 9 (see inset), the initial PLMA_194_‐PHEMA_2000_ nanoparticle dispersion is relatively turbid, with a transmittance of only 21% at 600 nm. In contrast, the acid‐swollen nanoparticles exhibit a significantly higher transmittance of 61%. After TEOS addition at 25°C, the absorbance briefly increases before progressively decreasing until around 40–50 min, at which point a limiting value of approximately 0.01 is attained. At this point, the sol‐gel reaction is likely to have reached completion. After 90 min, the resulting silica‐loaded nanoparticles are highly transparent, with a final transmittance of 98%. This is attributed to the fortuitous RI match between the hybrid PHEMA/silica nanoparticle cores and the continuous phase, which contains a mixture of PAO and ethanol.

**FIGURE 9:**
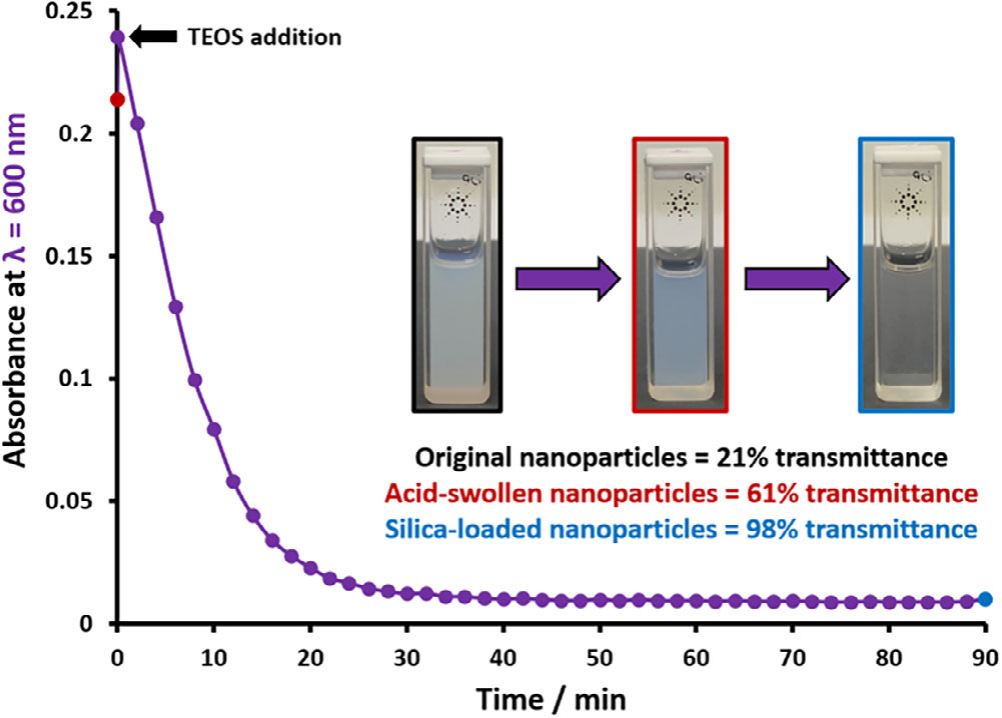
Absorbance (*λ* = 600 nm) versus time plot recorded using a visible absorption spectrophotometer during the silicification of a 10% *w*/*w* dispersion of acid‐swollen PLMA_194_‐PHEMA_2000_ nanoparticles in PAO at 25°C after addition of TEOS. Inset: digital photographs recorded for the original PLMA_194_‐PHEMA_2000_ nanoparticles (black), the acid‐swollen PLMA_194_‐PHEMA_2000_ nanoparticles (red), and the final silica‐loaded PLMA_194_‐PHEMA_2000_ nanoparticles (blue). Note that the latter dispersion is highly transparent (98% transmittance).

To more closely monitor the kinetics of the silicification reaction, a time‐resolved synchrotron SAXS experiment was also conducted. The corresponding series of *I*(*q*) versus *q* plots are presented in Figure 10a. TEOS was added to a 10% *w*/*w* dispersion of acid‐swollen PLMA_194_‐PHEMA_2000_ nanoparticles at 25°C, immediately shaken, then transferred into a flow‐through cell. Only minimal changes in the SAXS patterns were observed beyond 50 min, suggesting that the silicification reaction has reached completion within this timeframe. This observation is consistent with the visible absorption spectroscopy data shown in Figure 9. Multiple minima are observed in all SAXS patterns throughout the reaction, indicating controlled particle growth with no loss in colloidal stability. An additional structure factor at low *q* appears as silicification proceeds, indicating additional interparticle interactions within this relatively concentrated dispersion. Monitoring the evolution of the primary minimum over time enables the increase in nanoparticle core diameter to be calculated (see Figure 10b). After 90 min, the final volume‐average core diameter of the silica‐loaded nanoparticles was approximately 96 nm. This is consistent with DLS analysis of the final nanoparticles, which indicated a *z*‐average hydrodynamic diameter of 108 nm (DLS PDI = 0.03). TEM analysis of the final silica‐loaded nanoparticles confirmed a well‐defined morphology comprising uniform spheres (see inset shown in Figure 10b).

**FIGURE 10:**
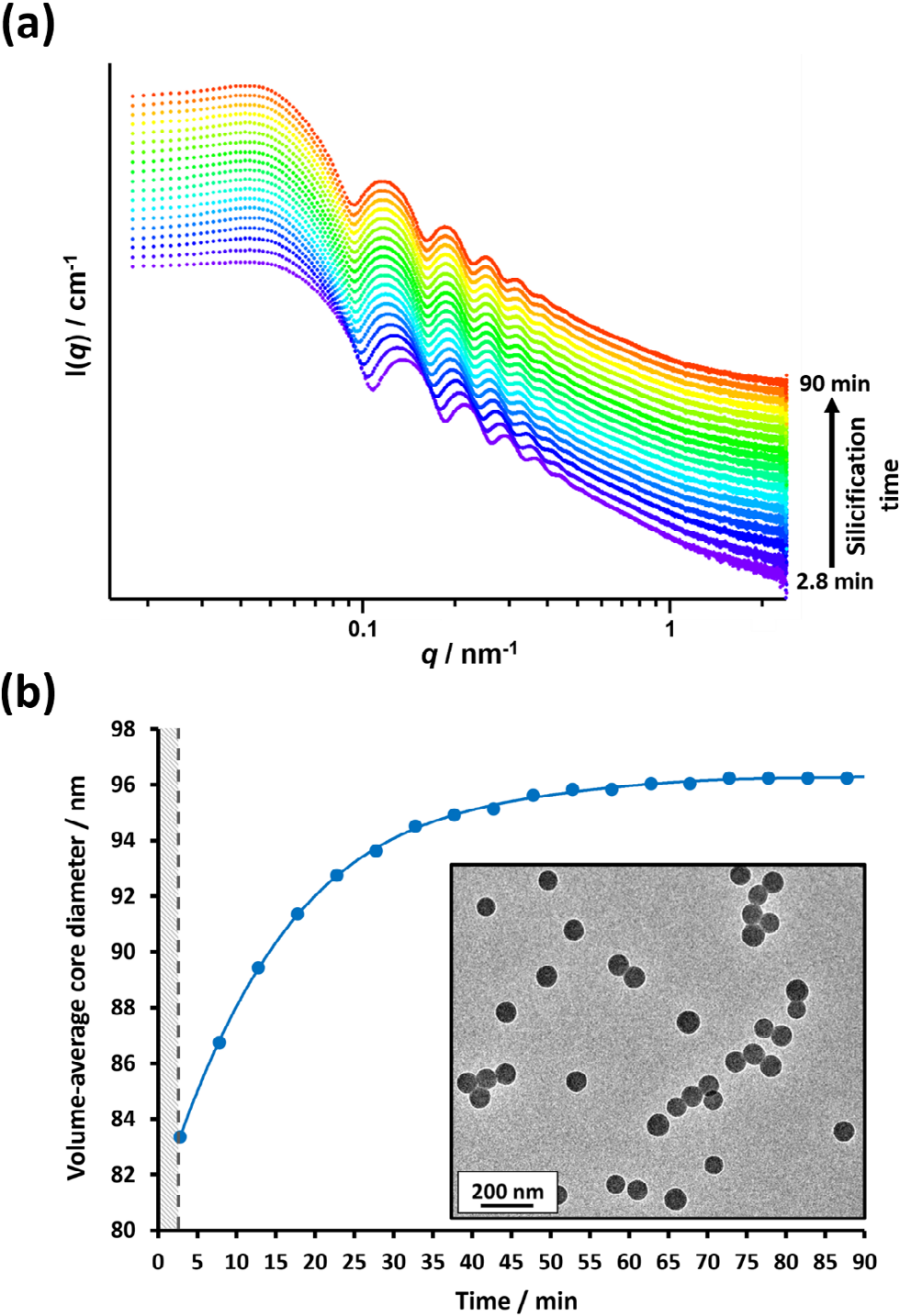
(a) Time‐resolved SAXS patterns recorded during the silicification of a 10% *w*/*w* dispersion of acid‐swollen PLMA_194_‐PHEMA_2000_ nanoparticles in PAO using TEOS at 25°C. For the sake of clarity, each SAXS pattern has been scaled by an arbitrary factor. (b) Evolution in the volume‐average core diameter versus reaction time for the same reaction (this kinetic experiment had a dead time of 2.8 min). Inset: representative TEM image recorded for a 0.1% *w*/*w* dispersion of the final silica‐loaded PLMA_194_‐PHEMA_2000_ nanoparticles.

### 2.5 Preparation of Titania‐Loaded PLMA_194_‐PHEMA_2000_ Nanoparticles

In this study, titanium *tert*‐butoxide was employed to prepare the titania‐loaded nanoparticles. However, initial experiments were unsuccessful owing to the high moisture sensitivity of this reagent. Hence, this oil‐soluble precursor was added dropwise over 10 min to the acid‐swollen PLMA_194_‐PHEMA_2000_ nanoparticles, which were maintained under a nitrogen atmosphere. Once in contact with the acidic solution, the titanium *tert*‐butoxide rapidly hydrolyses to form Ti(OH)_4_ and *tert*‐butanol [82, 83, 84, 85]. Subsequent condensation reactions lead to the formation of titania, which produces a turbid dispersion owing to its high RI.

Following titania formation, DLS analysis reveals an increase in the mean hydrodynamic nanoparticle diameter compared to that for the original or acid‐swollen nanoparticles (see Figure 11), while the relatively low DLS polydispersity suggests that minimal nanoparticle aggregation occurs. TEM analysis of the titania‐loaded nanoparticles (see Figure 11 inset) indicates a distinct change from a spherical morphology to framboidal nanoparticles (see Scheme S1). This suggests that titania is formed rapidly at the oil–water interface, before the titanium *tert*‐butoxide reagent can diffuse into the nanoparticle cores. The increase in DLS diameter is consistent with the corresponding SAXS patterns shown in Figure 12, where the primary minimum is shifted toward low *q*.

**FIGURE 11:**
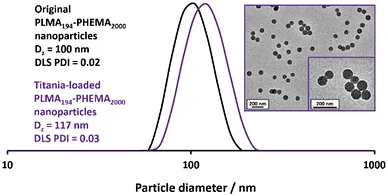
DLS particle size distributions recorded for 0.1% *w*/*w* dispersions of the original nanoparticles and titania‐loaded nanoparticles with the corresponding TEM image (inset: higher magnification image) obtained for the titania‐loaded nanoparticles.

**FIGURE 12:**
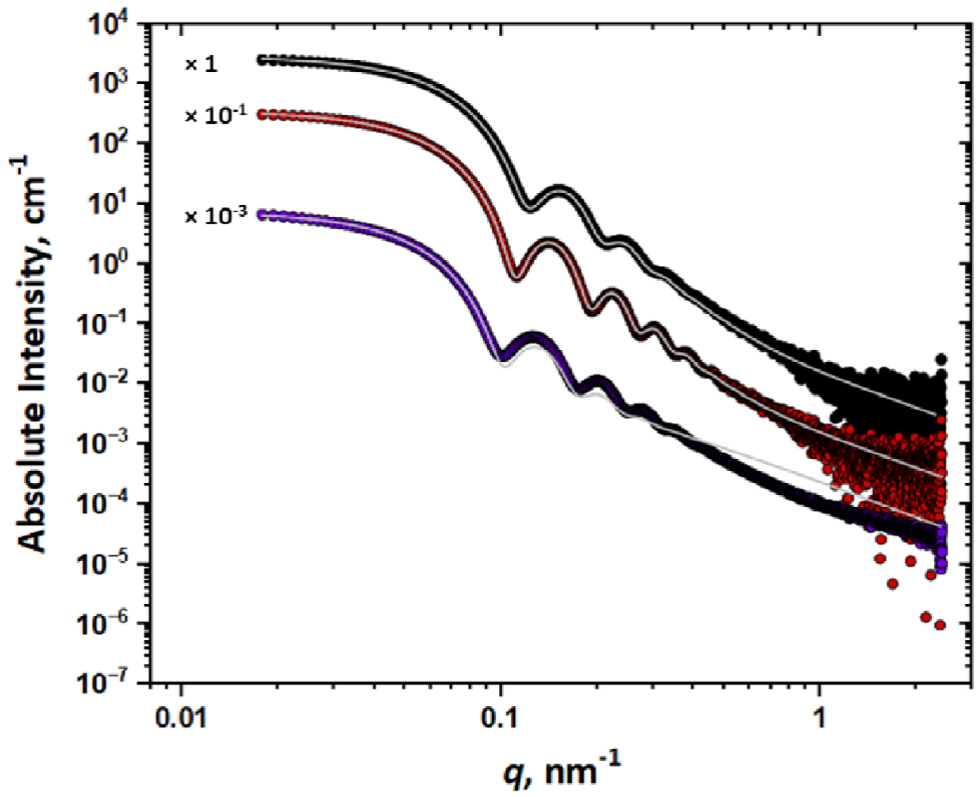
SAXS patterns recorded for 1.0% *w*/*w* dispersions of the original PLMA_194_‐PHEMA_2000_ nanoparticles (black), acid‐swollen nanoparticles (red), and titania‐loaded nanoparticles (purple) in PAO, where the first two curves have been fitted using a well‐known spherical micelle model (grey curves) [57], the latter fit for the titania‐loaded nanoparticles was obtained using a modified version of the blob model [71]. See the Supporting Information for details of the SAXS models. The SAXS patterns have been offset by an arbitary multiplication factor for clarity.

The modified scattering model used to fit the silica‐loaded nanoparticles did not produce a satisfactory fit for the SAXS pattern recorded for the titania‐loaded nanoparticles (see Figure 12). Even the best data fit deviated significantly from the experimental pattern over the whole *q* range and particularly at high *q*. Bearing in mind the relatively fast rate of hydrolysis of the oil‐soluble Ti(O*^t^* Bu)_4_ precursor, we postulated that the titania might be deposited in the form of a particulate shell around the nanoparticle core (see Scheme S1), rather than diffusing throughout the core prior to its hydrolysis, which appears to be the case for silica formation. Indeed, TEM analysis provides some evidence for this hypothesis (see Figure 11). Thus, a core–shell scattering model was also briefly examined when attempting to fit the SAXS pattern recorded for the titania‐loaded nanoparticles. However, this model cannot account for the diffuse nature of the coronal layer or the likely particulate morphology for the titania component. Hence, it is unlikely that this model would provide a satisfactory data fit to the whole SAXS pattern, particularly in the high *q* region. Nevertheless, given that the titania and PHEMA components dominate the overall x‐ray scattering, this core–shell model should still capture the main structural features of these titania‐loaded nanoparticles. Hence, it was applied to fit the SAXS pattern recorded for a 1.0% *w*/*w* dispersion of titania‐loaded PLMA_194_‐PHEMA_2000_ nanoparticles over a restricted *q* range (from *q* = 0.02 to *q* = 2.4 nm^−1^; see Figure S5). From solution densitometry measurements (see Figure S3), the overall nanoparticle density was determined to be 1.4707 g cm^−^^3^, which corresponds to a nanoparticle volume fraction of 0.0054. This volume fraction was used for the data fit and the scattering length density (SLD) for the surrounding medium (ξ_PAO_) was also fixed. This pragmatic approach produced a reasonably satisfactory fit to the scattering pattern (Figure S5). The SLDs calculated for the nanoparticle core (12.8 × 10^10^ cm^−2^) and shell (15.9 × 10^10^ cm^−2^) are comparable to the SLDs calculated for PHEMA (11.96 × 10^10^ cm^−2^) and TiO_2_ (17.76 × 10^10^ cm^−2^), respectively, which suggests that the nanoparticle cores mainly comprise these two components. Moreover, the core radius for such titania‐loaded nanoparticles is comparable to that calculated for the original PLMA_194_‐PHEMA_2000_ nanoparticles (Table S3). This is an important observation because it suggests that the titania is present as a shell surrounding non‐solvated PHEMA cores. Finally, SAXS analysis indicates that the mean thickness of this titania shell is approximately 64 Å, which is consistent with our TEM studies (see Figure 11). Interestingly, using the above core–shell model to fit SAXS patterns recorded for the silica‐loaded nanoparticles produced a very different result. In this case, the data fit returned an SLD of ∼ 13 × 10^10^ cm^−2^ for both the core and shell components, which lies between the SLD values calculated for silica and PHEMA. This suggests that this core–shell model is not physically realistic for the silica‐loaded nanoparticles.

TGA studies were performed on the dried titania‐loaded nanoparticles to determine their titania loading (see Figure 13). The incombustible titania content was 21.0% by mass, which corresponds to an overall yield of 96% based on the amount of soluble titania precursor used. FT‐IR spectroscopy analysis of the titania‐loaded nanoparticles prior to TGA (see Figure 14) revealed a very broad band at around 400–900 cm^−1^, which is absent in the FT‐IR spectrum of the original nanoparticles.

**FIGURE 13:**
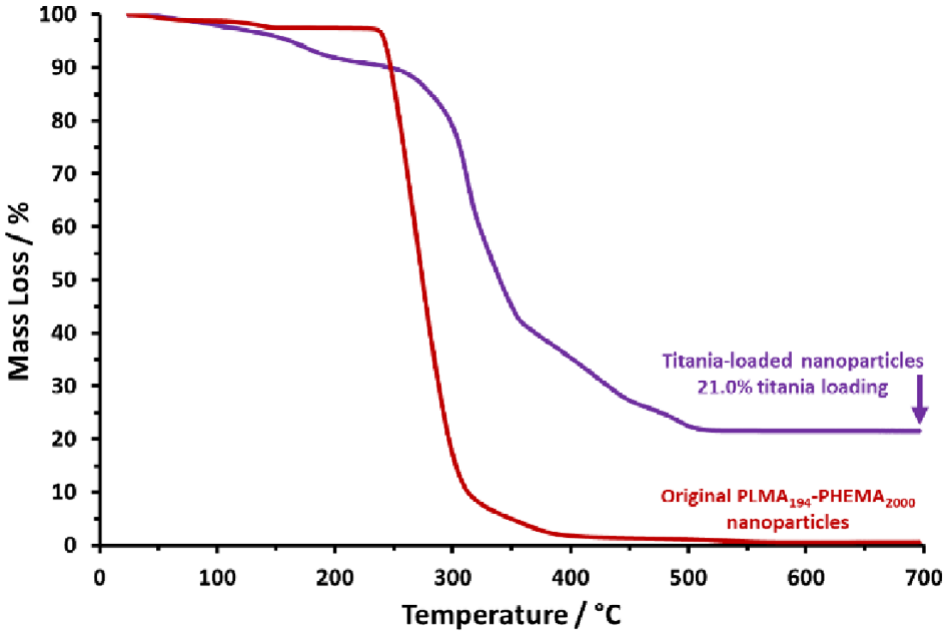
TGA curves recorded in air for the original PLMA_194_‐PHEMA_2000_ nanoparticles (red trace) and the titania‐loaded PLMA_194_‐PHEMA_2000_ nanoparticles (purple trace).

**FIGURE 14:**
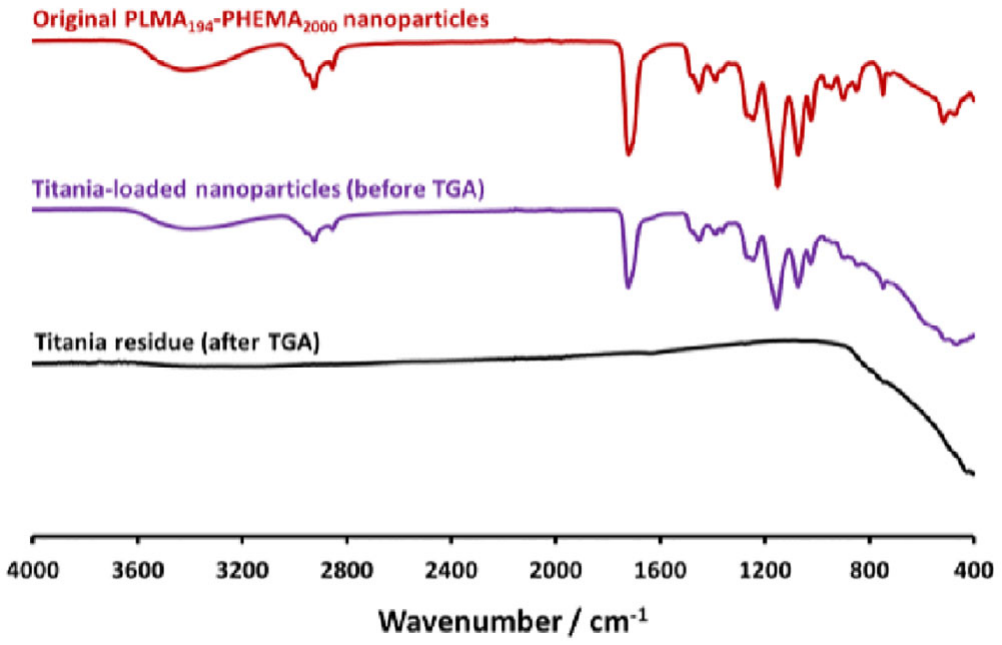
FT‐IR spectra recorded for the original PLMA_194_‐PHEMA_2000_ nanoparticles (red spectrum), titania‐loaded nanoparticles (purple spectrum), and the incombustible titania residue obtained after TGA analysis (black spectrum).

This feature is assigned to the Ti–O stretching vibration [86, 87, 88] and is also observed in the FT‐IR spectrum recorded for the TGA residue. Powder x‐ray diffraction (PXRD) analysis of the dried titania‐loaded nanoparticles was also conducted (Figure S2) but no distinct Bragg peaks corresponding to crystalline titania were observed. In principle, this might be because x‐ray scattering from the amorphous PLMA_194_‐PHEMA_2000_ component obscures the anatase/rutile crystalline peaks. Alternatively, it may be that only amorphous titania has been formed. The latter explanation is consistent with literature reports suggesting that thermal annealing is usually required to produce crystalline titania [89, 90, 91]. Moreover, it seems likely that generating titania within the nanoparticles in the presence of a relatively high volume fraction of reactive PHEMA chains is not conducive to the formation of highly ordered crystalline domains.

## 3 Conclusions

Acid‐swollen sterically‐stabilized block copolymer nanoparticles of around 100 nm diameter have been examined as nanoreactors for the in situ generation of either silica or titania in non‐polar media. These inorganic materials are efficiently produced within the lightly cross‐linked nanoparticle cores, with colloidal stability being retained at ≈12% *w*/*w* solids. The primary hydroxyl groups on the core‐forming PHEMA chains almost certainly participate in the sol‐gel chemistry, leading to highly cross‐linked nanocomposite nanoparticles. In the case of the silica‐loaded nanoparticles, the final morphology is spherical, silicification is essentially complete within 50 min at 25°C, the final colloidal dispersion is highly transparent, and the silica loading is around 17% by mass. In contrast, colloidal dispersions comprising the titania‐loaded nanoparticles are highly turbid and the titania loading within the nanoparticles is approximately 21% by mass. Moreover, the sol‐gel kinetics are significantly faster in this case, which leads to a distinctly framboidal core–shell morphology owing to a diffusion‐controlled interfacial reaction. In both cases, the inorganic component is amorphous rather than crystalline. Finally, in a closely related study we show that silica‐loaded nanoparticles can boost the thermal conductivity of the PAO oil [92].

## Author Contributions

**Priyanka Chohan**: investigation, methodology, validation, visualization, software, formal analysis, data curation, writing – original draft, writing – review and editing. **Andi Xie**: formal analysis, methodology, software. **Oleksandr O. Mykhaylyk**: methodology, software, validation, investigation, writing – original draft, writing – review and editing, formal analysis, supervision. **Steven P. Armes**: conceptualization, funding acquisition, writing – original draft, writing – review and editing, project administration, data curation, supervision, resources, methodology, formal analysis. **Giles M. Prentice**: funding acquisition, writing – review and editing, project administration, resources, supervision. **David J. Atkinson**: funding acquisition, writing – review and editing, project administration, resources, supervision. **Andrew J. Smith**: software, formal analysis, resources, investigation.

## Conflicts of Interest

The authors declare no conflicts of interest.

## Supporting information




**Supporting File**: anie74496‐sup‐0001‐SuppMat.docx.

## Data Availability

The data that support the findings of this study are available on request from the corresponding author. The data are not publicly available due to privacy or ethical restrictions.
