# Supplementary material for: Diblock Copolymer Nanoparticles Act as Nanoreactors for the Synthesis of Sterically‐Stabilized Inorganic Oxide Particles in Non‐polar Media
Source: Angew Chem Int Ed Engl. 2026 Sep 12;65(44):e4923622. doi: 10.1002/anie.4923622 (PMC13703897; doi:10.1002/anie.4923622)
Supplement: Supplementary file 1 [file ANIE-65-e4923622-s001.docx]

**Supporting Information for:**

***Diblock Copolymer Nanoparticles act as Nanoreactors for the Synthesis***

***of Sterically-Stabilized Inorganic Oxide Particles in Non-Polar Media***

Priyanka Chohan,^a^ Andi Xie,^a^ Oleksandr O. Mykhaylyk,^a^ Steven P. Armes,^a,^*

Giles M. Prentice,^b^ D. J. Atkinson^b^ and Andrew J. Smith^c^

[a] School of Mathematical and Physical Sciences, University of Sheﬃeld,

Sheffield, South Yorkshire, S3 7HF, UK

[b] Fluid Research, Castrol Technology Centre, Whitchurch Hill, Reading, RG8 7QR (UK)

[c] Diamond Light Source, Diamond House, Harwell Science and Innovation Campus,

Didcot, Oxford, OX11 0DE, UK

Table of Contents

[**Experimental**	S2](#_Toc76045799)

[**Table S1.** Summary of the DLS and TEM data obtained for a series of PLMA_194_-PHEMA_x_ nanoparticles prepared at 10% w/w solids in PAO under monomer-starved condtions	S8](#_Toc76045799)

[**Table S2.** Summary of the nanoparticle diameters obtained by TEM, SAXS and DLS for a series of PLMA_194_-PHEMA_x_ nanoparticles	S8](#_Toc76045799)

**Calculation of the PLMA_194_-PHEMA_2000_ nanoparticle mass increase after swelling using an aqueous solution of 0.3 M HCl**	S9

[**Figure S1.** *In situ* visible absorption spectra recorded for the reaction of aqueous HCl swollen PLMA_194_-PHEMA_2000_ nanoparticles with TEOS at 25 °C over 90 min	S10](#_Toc76045804)

**Figure S2.** PXRD pattern of dried titania-loaded PLMA_194_-PHEMA_2000_ nanoparticles	S10

**Solution densitometry measurements**	S11

**Figure S3.** Solution densitometry measurements for the original, acid-swollen, silica-loaded and titania-loaded PLMA_194_-PHEMA_2000_ nanoparticles	S12

**Figure S4.** Solution densitometry measurements for silica-in-water and titania-in-water suspensions	S14

[**Figure S5.** SAXS pattern recorded for the titania-loaded PLMA_194_-PHEMA_2000_ nanoparticles in PAO fitted using a core-shell model	S15](#_Toc76045804)

[**Scheme S1.** Schematic representation of the titania-loaded nanoparticles	S15](#_Toc76045804)

**Spherical micelle scattering model used for SAXS analysis**	S16

**Table S3.** Summary of structural parameters calculated from SAXS analysis of the	PLMA_194_-PHEMA_2000_ nanoparticles	S20

**References**	S20

**Experimental**

**Materials**

Lauryl methacrylate (LMA; 96%) was purchased from Sigma-Aldrich (UK) and filtered through basic alumina to remove monomethyl ether hydroquinone (MEHQ) inhibitor prior to use. 2-Hydroxyethyl methacrylate (HEMA, triply distilled ULTRA grade; 0.1 mol% dimethacrylate impurity) was kindly provided by GEO Specialty Chemicals (Hythe, UK) and was used as received. Cumyl dithiobenzoate (CDB; 99%), deuterium oxide (D_2_O), deuterium chloride (DCl), tetraethyl orthosilicate (TEOS; 98%), 2,6-di-*tert*-butyl-4-methyl-phenol (BHT; 99%), ruthenium(IV) oxide hydrate and sodium periodate (99.8%) were purchased from Sigma-Aldrich (UK). Hydrochloric acid (HCl; 37%), *n*-dodecane, *d*_5_-pyridine and titanium(IV) *tert*-butoxide (98%) were purchased from Thermo Fisher Scientific (UK). *d*_2_-Dichloromethane (CD_2_Cl_2_) and *d*_26_-dodecane were purchased from Cambridge Isotope Laboratory (USA). Tetrahydrofuran (THF; HPLC grade) and triethylamine (TEA; 99%) were obtained from VWR Chemicals (UK) and *tert*-butyl peroxy-2-ethylhexanoate (T21s) initiator was supplied by AkzoNobel (The Netherlands). The poly(α-olefin) oil (PAO; Durasyn 162) was provided by BP Chemicals Ltd. (Pangbourne, UK) and *n*-heptane was purchased from Alfa Aesar (UK). AngstromSphere monodispersed silica powder was obtained from Fiber Optic Centre Inc. (Massachusetts, USA). Deionized water (resistivity = 15 MΩ cm) was obtained from an Elgastat Option 3A water purification system.

**Synthesis of PLMA_194_ precursor via RAFT solution polymerization of LMA in PAO**

A PLMA_194_ homopolymer was prepared at 80% w/w solids by adapting a previously reported protocol.^[1]^ The synthesis of this precursor was conducted as follows. LMA (59.77 g; 0.235 mol), CDB (0.32 g; 1.175 mmol; target DP = 200), T21s initiator (84.7 mg; 0.392 mmol; CDB/T21s molar ratio = 3.0; dissolved at 10% v/v in 0.678 g PAO) and PAO (14.36 g) were weighed into a 100 mL round-bottomed flask containing a magnetic flea, which was sealed using a rubber septum. The resulting solution was purged with nitrogen gas for 30 min. Then the sealed flask was immersed in a preheated oil bath at 90 °C to initiate the LMA polymerization and the reaction mixture was magnetically stirred for 15 h. A final LMA conversion of 97% was determined by ^1^H NMR spectroscopy studies in CD_2_Cl_2_ by comparing the integrated vinyl monomer signals at 5.6 ppm and 6.1 ppm to the two oxymethylene protons assigned to PLMA at 3.8–4.1 ppm. Accordingly, the mean DP of this crude PLMA precursor was calculated to be approximately 194. THF GPC analysis using a refractive index detector and a series of near-monodisperse poly(methyl methacrylate) calibration standards indicated an *M*_n_ of 40 100 g mol^-1^ and an *M*_w_/*M*_n_ of 1.16.

**Synthesis of PLMA_194_-PHEMA_x_ nanoparticles via RAFT dispersion polymerization of HEMA**

PLMA_194_-PHEMA_x_ nanoparticles (where x = 1000 – 3000) were targeted at 10% w/w solids *via* monomer-starved addition of HEMA, as previously reported.^[2]^ A typical synthesis of PLMA_194_-PHEMA_2000_ nanoparticles was conducted as follows. An as-synthesized PLMA_194_ precursor (1.875 g, 80% w/w solids; 30.2 μmol), T21s initiator (2.18 mg; 10.1 μmol; dissolved at 10% v/v in 0.0174 g PAO) and PAO (83.94 g) were weighed into a 250 mL round-bottomed flask equipped with a magnetic flea. HEMA monomer (8.5 g) was added to a separate 14 mL glass vial. Both vials were then sealed and purged with nitrogen gas for 30 min. Degassed HEMA monomer (7.87 g; 60.4 mmol; target DP = 2000) was drawn into a degassed 10 mL syringe, which was then placed into a motorized Aladdin AL-1000 syringe pump. The flask containing the PLMA_194_ precursor was immersed in a preheated oil bath set at 90 °C, and HEMA monomer was added dropwise over 2.0 h at a rate of 3.67 mL h^-1^ with continuous magnetic stirring. After complete addition of this monomer, the polymerization was allowed to proceed for a further 15 h at 90 °C. A final HEMA conversion of more than 99% (i.e. no detectable monomer vinyl signals) was determined *via* ^1^H NMR analysis in *d*_5_-pyridine, indicating a PHEMA DP of approximately 2000. Similarly, PLMA_194_-PHEMA_1000_, PLMA_194_-PHEMA_1500_ and PLMA_194_-PHEMA_3000_ nanoparticles were also targeted at 10% w/w solids, with the addition of HEMA monomer taking place over 1.0 h, 1.5 h and 3.0 h, respectively. Final HEMA conversions of at least 97% were achieved in each case.

**Swelling of PLMA_194_-PHEMA_2000_ nanoparticles using aqueous HCl**

A 10% w/w dispersion of PLMA_194_-PHEMA_2000_ nanoparticles prepared under monomer-starved conditions in PAO (2.0 g; corresponding to 0.168 g PHEMA_2000_) and an aqueous solution of 0.3 M HCl (0.0504 g, 30% w/w based on the PHEMA_2000_ mass; 2.80 mmol water) were placed in a 7 mL glass vial, sealed and stirred at 1000 rpm for three days at 25 °C to obtain acid-swollen PLMA_194_-PHEMA_2000_ nanoparticles for analysis by DLS, SAXS and TEM.

**Swelling of PLMA_194_-PHEMA_2000_ nanoparticles redispersed in *d*_26_-dodecane using a DCl/D_2_O mixture**

A 10% w/w dispersion of PLMA_194_-PHEMA_2000_ nanoparticles dispersed in PAO (1.00 g; corresponding to 0.084 g PHEMA_2000_) was placed in a 1.5 mL microfuge tube and centrifuged (using a Heraeus Biofuge Pico D-37520) at 13,000 rpm for 3 h. The supernatant (0.816 g) was then removed and the sedimented nanoparticles were redispersed in *d*_26_-dodecane (0.816 g) for ^1^H NMR analysis. A sample of PLMA_194_-PHEMA_2000_ nanoparticles in *d*_26_-dodecane (0.500 g) was weighed into a 7 mL glass vial containing a 0.3 M DCl/D_2_O solution (0.0126 g; 30% w/w based on the PHEMA_2000_ mass; 0.629 mmol) and stirred at 1000 rpm for three days at 25 °C. ^1^H NMR analysis was then conducted on the DCl/D_2_O-swollen nanoparticles in *d*_26_-dodecane.

**Preparation of silica-loaded PLMA_194_-PHEMA_2000_ nanoparticles**

TEOS (0.146 g; 0.699 mmol) was added *via* micropipette to a 7 mL glass vial containing aqueous acid-swollen PLMA_194_-PHEMA_2000_ nanoparticles dispersed in PAO (2.05 g). The vial was shaken for 30 s before allowing to stand unstirred for 16 h at 25 °C.

**Preparation of titania-loaded PLMA_194_-PHEMA_2000_ nanoparticles**

A 7 mL glass vial containing aqueous acid-swollen PLMA_194_-PHEMA_2000_ nanoparticles dispersed in PAO (2.05 g) was sealed using a rubber septum before replacing the air with nitrogen gas (5 min purge). A 1 mL syringe was degassed, filled with titanium *tert*-butoxide (0.238 g; 0.699 mmol), and then placed into a motorized Aladdin AL-1000 syringe pump. The titanium *tert*-butoxide precursor was added to the dispersion under nitrogen at 25 °C over a 10 min period at a rate of 26.76 μL min^-1^ with magnetic stirring at 825 rpm. The rubber septum was replaced with a screw-on cap and the reaction mixture was magnetically stirred for a further 16 h at 25 °C.

**Preparation of dried inorganic oxide-loaded PLMA_194_-PHEMA_2000_ nanoparticles**

The silica-loaded and titania-loaded nanoparticles were purified by centrifugation. First, the dispersions of inorganic oxide-loaded nanoparticles dispersed in PAO (0.80 g) were placed in 1.5 mL microfuge tubes. Pairs of tubes were mass-balanced before being placed in a microcentrifuge (Heraeus Biofuge Pico D-37520) and centrifugation was conducted for 60 min at 13,000 rpm. In each case, the supernatant was decanted and the sedimented nanoparticles were redispersed in *n*-heptane (0.50 mL) prior to centrifugation for 20 min at 13,000 rpm. This centrifugation-redispersion was repeated once more for both samples. Finally, the moist nanoparticle sediments were placed in a vacuum oven and dried at 40 °C for three days. The original (un-swollen) PLMA_194_-PHEMA_2000_ nanoparticles were also dried following the same protocol.

**Characterization Techniques**

**^1^H NMR Spectroscopy**

^1^H NMR spectra were recorded at 298 K in either CD_2_Cl_2_, *d*_5_-pyridine or *d*_26_-dodecane using a 400 MHz Bruker Avance spectrometer. Typically, 64 scans were averaged per spectrum.

**Gel Permeation Chromatography (GPC)**

THF GPC was used to assess the molecular weight distribution (MWD) of the PLMA_194_ precursor. The instrument comprised an Agilent 1260 GPC system and two Agilent PL gel 5 µm Mixed C columns, which were connected in series with a guard column and a 1260 Infinity II refractive index detector. The eluent contained BHT (1.25 g) and TEA (50 mL) in HPLC-grade THF (2500 mL), the operating temperature was 30 °C and the flow rate was 1.0 mL min^-1^. A series of nine near-monodisperse poly(methyl methacrylate) standards (with *M*_p_ values ranging from 7,290 to 2,210,000 g mol^-1^) were used for calibration.

**Dynamic Light Scattering (DLS)**

DLS studies were conducted using a Zetasizer Nano ZS instrument (Malvern Instruments, UK) at a ﬁxed scattering angle of 173°. The copolymer dispersions were diluted to 0.10% w/w using *n*-dodecane prior to light scattering studies at 25 °C. The z-average hydrodynamic diameter and DLS polydispersity were calculated by cumulant analysis of the experimental correlation function using Dispersion Technology Software version 6.20 and assuming that the Stokes-Einstein equation is valid. Data were averaged over three consecutive runs comprising ten measurements per run. Depending on the nature of the nanoparticle cores, the literature refractive indices for dry PHEMA (1.51), fully water-swollen PHEMA (1.44) and silica (1.46) were used for DLS analysis.

**Transmission Electron Microscopy (TEM)**

TEM studies were conducted using a JEOL JEM-120i electron microscope operating at 120 kV and equipped with a SightSKY 19 megapixel camera. A single droplet of a 0.10% w/w copolymer dispersion diluted in *n*-dodecane was placed on a carbon-coated copper grid and air-dried for 90 min, prior to exposure to ruthenium(VIII) oxide vapor for 7 min at 20 °C.^[3]^ This heavy metal compound acted as a positive stain to improve electron contrast and was prepared as follows. Ruthenium(IV) oxide (0.30 g) was added to water (50 g) to form a black slurry; addition of sodium periodate (2.0 g) with continuous stirring produced a yellow solution of ruthenium(VIII) oxide within 1 min at 20 °C.

**Fourier-Transform Infrared (FTIR) Spectroscopy**

FTIR spectra were recorded from 4000 to 400 cm^-1^ using a Perkin Elmer Spectrum Two instrument with the Diamond Attenuated Total Reflectance (ATR) accessory under ambient conditions. Typically, eight scans were averaged per spectrum and the spectral resolution was 4 cm^-1^.

**Thermogravimetric Analysis (TGA)**

Mass loss curves were recorded for the dried precursor PLMA_194_-PHEMA_2000_ nanoparticles and the inorganic oxide-loaded PLMA_194_-PHEMA_2000_ nanoparticles using a Q500 TA TGA instrument. Samples were heated under air from 20 °C to 700 °C at a heating rate of 10 °C min^-1^.

**Visible Absorption Spectroscopy**

Visible absorption spectra were recorded using an Agilent Cary 3500 Multicell UV-Vis Spectrophotometer equipped with a 250 Hz xenon flash lamp and Agilent Cary UV workstation 1.3.4 software. Absorption spectra were recorded between 400 and 800 nm at 25 °C using a Multicell Peltier UV-Vis detector module with an averaging time of 0.100 s and a spectral bandwidth of 2 nm. Prior to both static and *in situ* measurements, a baseline spectrum for the PAO was recorded at 25 °C. An *in situ* silicification reaction was monitored over 90 min with spectra being recorded every 2 min and the set-up was as follows. A 10% w/w dispersion of acid-swollen PLMA_194_-PHEMA_2000_ nanoparticles (2.05 g) was placed into a 10 mm quartz cuvette and an initial absorption spectrum was recorded (using PAO as a reference). TEOS (0.146 g; 0.699 mmol) was then added *via* micropipette and the cuvette was shaken for 30 s before being placed into the spectrophotometer and immediately starting the acquisition of spectra.

**Powder X-Ray Diffraction (PXRD)**

Powder XRD analysis was performed on the dried titania-loaded PLMA_194_-PHEMA_2000_ nanoparticles using Cu-Kα radiation (40 kV, 40 mA) on a Bruker D8 ADVANCE X-ray powder diffractometer equipped with a motorized divergence slit for Bragg-Brentano geometry and a high-resolution energy-dispersive Lynxeye XE detector.

**Solution densitometry**

Solution densities (± 0.000005 g cm^-3^) were determined using an Anton Paar DMA 5000 densitometer operating at 25 °C. Known concentrations of the as-prepared, acid-swollen, silica-loaded or titania-loaded PLMA_194_-PHEMA_2000_ nanoparticle dispersions, as well as silica-in-water and titania-in-water suspensions (1.0 mL) were prepared before being injected in turn into a U-shaped borosilicate glass tube. After allowing 5 min for temperature equilibration, the solution density ($\rho$) of each sample was determined (see **Figures S3a** and **S4**) in the Supporting Information. Such data were used to calculate the X-ray scattering length density (SLD) in each case.

**Small-angle X-ray scattering (SAXS)**

SAXS experiments were conducted at station I22 of the Diamond Light Source (Didcot, UK) using monochromatic X-ray radiation (*λ* = 0.1 nm; *q* range from 0.018 to 2.4 nm^-1^, where *q* is the length of the scattering vector and *θ* is one-half of the scattering angle, such that *q* = 4πsin*θ*/*λ*) and a Pilatus 2M two-dimensional (2D) hybrid pixel detector (Dectris, Switzerland). The samples comprised four 1.0% w/w dispersions of PLMA_194_-PHEMA_980-2910_ nanoparticles in PAO, acid-swollen PLMA_194_-PHEMA_2000_ nanoparticles (30% w/w aqueous 0.3 M HCl based on the PHEMA mass), silica-loaded PLMA_194_-PHEMA_2000_ nanoparticles, and titania-loaded PLMA_194_-PHEMA_2000_ nanoparticles. A flow-through glass capillary of 1.79 mm diameter was used as a sample holder. Two-dimensional scattering patterns were integrated to give one-dimensional scattering curves and calibrated to absolute intensity (pure water was used as a scattering reference standard) using Dawn software package^[4]^ provided by the beamline. SAXS data processing (background subtraction) and further analysis (SAXS model fits to scattering patterns) were performed using Irena SAS macro for Igor Pro.^[5]^ A time-resolved SAXS study was also performed at station I22. In this case, the silicification of a 10% w/w dispersion of aqueous acid-swollen PLMA_194_-PHEMA_2000_ nanoparticles was conducted. TEOS (0.146 g; 0.699 mmol) was added to 10% w/w dispersion of pre-prepared acid-swollen PLMA_194_-PHEMA_2000_ nanoparticles (2.05 g) in a 7 mL glass vial, which was then shaken for 30 s. Using a 5 mL syringe, this reaction mixture was then transferred into a 1.32 mm diameter flow-through cell set at 25 °C. The dead time for this setup was estimated to be 2.8 min, which corresponds to the time taken to record the first SAXS pattern after mixing the two reactants.

**Table S1.** Summary of the DLS and TEM data obtained for a series of PLMA_194_-PHEMA_x_ nanoparticles prepared at 10% w/w solids in PAO under monomer-starved conditions using a syringe pump.

| **Target Composition** | **Time for HEMA addition / h** | **DLS** | | **TEM Morphology** |
| --- | --- | --- | --- | --- |
|  |  | **D_z_ / nm** | **PDI** |  |
| PLMA_194_-PHEMA_1000_ | 1 | 92 | 0.01 | Spheres |
| PLMA_194_-PHEMA_1500_ | 1.5 | 95 | 0.01 | Spheres |
| PLMA_194_-PHEMA_2000_ | 2 | 100 | 0.02 | Spheres |
| PLMA_194_-PHEMA_3000_ | 3 | 106 | 0.01 | Spheres |

**Table S2.** Summary of the nanoparticle diameters obtained by TEM, SAXS and DLS analysis for a series of PLMA_194_-PHEMA_x_ nanoparticles prepared at 10% w/w solids in PAO.^$^

| **Target copolymer composition** | **TEM^a^**  **number-average diameter / nm** | **SAXS^b^**  **volume-average diameter / nm** | **DLS^c^** | |
| --- | --- | --- | --- | --- |
|  |  |  | **D_z_ / nm** | **PDI** |
| PLMA_194_-PHEMA_1000_ | 63 | 59 | 92 | 0.01 |
| PLMA_194_-PHEMA_1500_ | 67 | 64 | 95 | 0.01 |
| PLMA_194_-PHEMA_2000_ | 78 | 73 | 100 | 0.02 |
| PLMA_194_-PHEMA_3000_ | 84 | 81 | 106 | 0.01 |

a. 100 nanoparticles analyzed per sample.

b. Calculated using the well-known spherical micelle model reported by J. S. Pedersen et al. (see ref. 6).

c. Hydrodynamic z-average diameter (D_z_) calculated using the Stokes-Einstein equation.

$. The estimated TEM number-average diameter is always higher than the volume-average diameter reported by SAXS. This is not mathematically possible and simply indicates that our analysis of 100 nanoparticles is insufficient to be statistically reliable. In contrast, the SAXS and DLS data are averaged over many millions of nanoparticles and are considered to be statistically robust.

**Calculation of the PLMA_194_-PHEMA_2000_ nanoparticle mass increase after swelling by 30% w/w (based on the mass of the PHEMA cores) using an aqueous solution of 0.3 M HCl**

The primary minimum ($q$*_min_*) in the SAXS patterns of the original nanoparticles and aqueous HCl swollen nanoparticles were used to calculate the mean particle radii ($R₁$ and $R₂$) using the equation:^[7]^

$R₁‚₂$= 4.49/$q$*_min_*	(S1)

The mass of PHEMA per particle ($m₁$) was calculated using the density of PHEMA ($\rho₁$), where $\rho₁$ = 1.28 g cm^-3^ (as measured by helium pycnometry and solution densitometry) and the volume of the PHEMA core ($V₁$):

$m₁=\rho₁V₁= \rho₁\frac{4}{3}\pi R₁^{3}$	(S2)

The volume of the swollen PHEMA cores ($V₂$) can be also be calculated:

$V₂= \frac{4}{3}\pi R₂^{3}$	(S3)

Assuming the increase in particle size is solely due to the uptake of aqueous HCl, the total volume of aqueous HCl up taken ($V₃$) can be calculated:

$V₃=V₂- V₁$	(S4)

Using $\rho₂$ = 1.002 g cm^-3^ for the density of 0.3 M HCl, the mass of aqueous HCl per particle ($m₂$) can be calculated:

$m₂=\rho₂V₃$	(S5)

Lastly, the % mass increase per PLMA_194_-PHEMA_2000_ particle after swelling ($m₃$) can be calculated:

$m₃=\frac{m₂}{m₁}\times100$	(S6)

**Figure S1.** *In situ* visible absorption spectra recorded at 25 °C for the reaction of a 10% w/w dispersion of aqueous HCl swollen PLMA_194_-PHEMA_2000_ nanoparticles in PAO with TEOS over 90 min. As the reaction progresses, the absorbance of the dispersion decreases as the TEOS is converted to silica within the nanoparticle cores, leading to a fortuitous match in the refractive index of the PHEMA/silica cores and the continuous phase at 25 °C.

**Figure S2.** Powder X-ray diffraction (PXRD) pattern of the dried titania-loaded PLMA_194_-PHEMA_2000_ nanoparticles. No distinct Bragg peaks corresponding to crystalline titania are observed. This is either due to the amorphous PLMA_194_-PHEMA_2000_ polymer obscuring the anatase/rutile peaks or because only amorphous titania has been formed, where the latter is more likely the case.

**Solution densitometry measurements**

**Calculation of original, acid-swollen, silica-loaded and titania-loaded PLMA_194_-PHEMA_2000_ nanoparticle densities**

The densities of the original PLMA_194_-PHEMA_2000_ nanoparticles, 0.3 M HCl swollen PLMA_194_-PHEMA_2000_ nanoparticles and subsequent silica-loaded and titania-loaded nanoparticles were calculated by solution densitometry. This involved measuring the density of a range of each dispersion at solid concentrations between 0.2 – 1.0% w/w in PAO (see **Figure S3a**). Since the relationship between the solution density and weight fraction is non-linear, conversion to volume fraction (using a PAO density, $\rho_{\mathrm{PAO}}$ = 0.7907 g cm^-3^) is required for extrapolation to 100% to obtain the solid-state density of the nanoparticles in each case (**Figures S3b-e**).

**Figure S3.** (a) Solution mass densities measured at 25 °C for the original PLMA_194_-PHEMA_2000_ nanoparticles (blue), 0.3 M HCl swollen PLMA_194_-PHEMA_2000_ nanoparticles (red), silica-loaded PLMA_194_-PHEMA_2000_ nanoparticles (green) and titania-loaded PLMA_194_-PHEMA_2000_ nanoparticles (purple) at solid concentrations between 0.2 – 1% w/w. Dispersion density vs. volume fraction of (b) PLMA_194_-PHEMA_2000_ nanoparticles, (c) 0.3 M HCl swollen PLMA_194_-PHEMA_2000_ nanoparticles, (d) silica-loaded PLMA_194_-PHEMA_2000_ nanoparticles and (e) titania-loaded PLMA_194_-PHEMA_2000_ nanoparticles.

**Calculation of PHEMA block density**

In order to determine the X-ray scattering length density contrasts for the PLMA and PHEMA blocks needed for SAXS analysis, the density of each block was determined using solution densitometry. Using a known PLMA density ($\rho_{\mathrm{PLMA}}$) of 0.921 g cm^-3^,^[2]^ the PHEMA block density ($\rho_{\mathrm{PHEMA}}$) can be calculated by measuring the density of a series of PLMA_194_-PHEMA_2000_ nanoparticles at copolymer concentrations between 0.2 – 1.0% w/w, as previously mentioned. A PLMA_194_-PHEMA_2000_ copolymer density ($\rho_{PLMA-PHEMA}$) of 1.211 g cm^-3^ was determined at 25 °C.

Next, the relative mass fractions of the PLMA and PHEMA blocks were determined using the PLMA and PHEMA degree of polymerization (DP) and the corresponding monomer molecular weights (MW):

$Mass PLMA \left( M_{PLMA} \right)=PLMA DP \times{MW}_{LMA}$	(S7)

$Mass PHEMA \left( M_{PHEMA} \right)=PHEMA DP \times{MW}_{HEMA}$	(S8)

$Total Mass \left( M_{Total} \right)= M_{PLMA}+ M_{PHEMA}$	(S9)

$Mass Fraction PLMA \left( W_{PLMA} \right)= M_{PLMA} / M_{Total}$	(S10)

$Mass Fraction PHEMA \left( W_{PHEMA} \right)= M_{PHEMA} / M_{Total}$	(S11)

Using the specific volume mixing rule then allows the $\rho_{\mathrm{PHEMA}}$ to be calculated as 1.286 g cm^-3^:

$\frac{1}{\rho_{PLMA-PHEMA}}= \frac{W_{PLMA}}{\rho_{PLMA}} + \frac{W_{PHEMA}}{\rho_{PHEMA}}$	(S12)

**Calculation of silica and titania density**

The density of silica and titania formed within the nanoparticles was estimated by solution densitometry. This involved preparing silica and titania in 0.3 M HCl solution in the absence of the PLMA_194_-PHEMA_2000_ nanoparticles.

The silica was prepared in a 7 mL glass vial by stirring 0.3 M HCl (1.00 g; 55.5 mmol) with tetraethyl orthosilicate (2.8910 g; 13.9 mmol) at 25 °C for 16 h. Similarly, the titania was prepared by stirring 0.3 M HCl (0.2 g; 11.1 mmol) with titanium-*tert*-butoxide (0.945 g; 2.78 mmol) in a 7 mL glass vial at 25 °C for 16 h. Centrifugation (30 min, 13,000 rpm) was then used to purify the resulting silica and titania before drying each sample in a vacuum oven set at 40 °C for 3 days.

A series of aqueous suspensions of silica and titania were then prepared at 0.0625 – 1.0% w/w and solution densitometry measurements were conducted at 25 °C (see **Figure S4**). More specifically, the solution density data were fitted using equation S13^[8]^ and then extrapolated to 100% to determine the solid-state density of silica (or titania).

$\rho_{susp}= \frac{1}{\frac{{1 - C}_{tot}}{\rho_{w}} + \frac{C_{tot}}{\rho_{{XO}_{2}}}}$	(S13)

Where $\rho_{susp}$ is the density of the suspension, $C_{tot}$ is the total solids concentration, $\rho_{{XO}_{2}}$ is the density of either silica or titania, and $\rho_{w}$ is the mass density of water at 25 °C.

**Figure S4.** Solution densities determined at 25 °C for a series of aqueous suspensions of (a) silica and (b) titania at concentrations ranging between 0.0625 and 1.0% w/w. Fitting these data enabled calculation of the solid-state density of each inorganic oxide formed within the PLMA_194_-PHEMA_2000_ nanoparticles, which is required for SAXS analysis (see below).^[8]^

**Figure S5.** SAXS pattern recorded for a 1.0% w/w dispersion of the titania-loaded PLMA_194_-PHEMA_2000_ nanoparticles in PAO fitted using a core-shell model (grey curve).^[6]^ This scattering model provides a reasonably satisfactory fit at low and intermediate q but a poor fit at high q.

**Scheme S1.** Schematic representation of the two possible morphologies for the titania-loaded nanoparticles. Left-hand side with titania exclusively formed within the nanoparticle core and the right-hand side with titania nanoparticles formed at the PAO oil-water interface.

**Spherical micelle scattering model used for SAXS analysis**

The scattering intensity of a single population of non-interacting particles can be expressed as:

$I(q)=N\int_{0}^{\infty} ...\int_{0}^{\infty} F(q,r_{1},...,r_{k})^{2}\Psi(r_{1},...,r_{k})dr_{1}...dr_{k}$ (S14)

where $F(q,r_{1},...,r_{k})$ is the particle form factor expressed as a function of *r*_1_,...,*r*_k_ parameters, $\Psi(r_{1},...,r_{k})$ is the distribution function of the form factor parameters and *N* is the particle number density per unit volume. For a population of spherical diblock copolymer micelles, the form factor in eq. S14 can be expressed as:^[9,10]^

$\begin{matrix} F_{\text{mic}}(q,r_{1})=N_{\text{agg}}^{2}(r_{1})\beta_{\text{s}}^{2}F_{\text{s}}(q,r_{1})+N_{\text{agg}}(r_{1})\beta_{\text{c}}^{2}F_{\text{c}}(q,R_{\text{g}})+ \\ +N_{\text{agg}}(r_{1})[N_{\text{agg}}(r_{1})-1]\beta_{\text{c}}^{2}A_{\text{c}}^{2}(q,r_{1})+2N_{\text{agg}}^{2}(r_{1})\beta_{\text{s}}\beta_{\text{c}}A_{\text{s}}(q,r_{1})A_{\text{c}}(q,r_{1}) \end{matrix}$ (S15)

The micelle core radius, *r*_1_, has a dispersity that can be described by a Gaussian distribution:

$\Psi(r_{1})=\frac{1}{\sqrt{2\pi\sigma_{R\text{s}}^{2}}}e^{-\frac{(r_{1}-R_{\text{s}})^{2}}{2\sigma_{R\text{s}}^{2}}}$ (S16)

where *R*_s_ is the mean spherical micelle core radius and *σ_R_*_s_ is its standard deviation. *R_g_* in eq. S15 is the radius of gyration of the corona block (PLMA), and $\beta_{\text{s}}=V_{\text{s}}(\xi_{\text{s}}-\xi_{\text{sol}})$ and $\beta_{\text{c}}=V_{\text{c}}(\xi_{\text{c}}-\xi_{\text{sol}})$are the X-ray scattering length density contrasts for the PHEMA core block and the PLMA corona block, respectively. *ξ*_s_, *ξ*_c_, and *ξ*_sol_ are X-ray scattering length densities for the core block (*ξ*_PHEMA_ = 11.96 × 10^10^ cm^-2^), the corona block (*ξ*_PLMA_ = 8.72 × 10^10^ cm^-2^) and the solvent (*ξ*_PAO_ = 7.69 × 10^10^ cm^-2^), respectively. *V*_s_ and *V*_c_ are the volumes of the core block and the corona block, respectively. The core block and the corona block volumes can be calculated using the equation $V=\frac{M_{\text{w}}}{N_{\text{A}}\rho}$, given knowledge of the mean number of repeat units in the core block and the corona block, the molecular weight of each repeat unit (254.3 kg mol^-1^ for LMA and 130.1 kg mol^-1^ for HEMA), and the mass density of each block (*ρ*_PLMA_ = 0.921 g cm^-3^, as measured by solution densitometry measurements for a range of PLMA concentrations dissolved in *n*-dodecane and *ρ*_PHEMA_ = 1.286 g cm^-3^, as calculated from solution densitometry measurements for a series of PLMA_194_-PHEMA_2000_ dispersions in PAO at various copolymer concentrations using *ρ*_PLMA_) and the density of PAO (C_20_H_42_, *ρ*_PAO_ = 0.7907 g cm^-3^). It is perhaps noteworthy that the *ρ*_PHEMA_ value calculated from solution densitometry is consistent with that determined by helium pycnometry (1.28 g cm^-3^).

The mean micelle aggregation number in eq. S15 is given by$N_{\text{agg}}(r_{1})=(1-x_{\text{sol}})\frac{4\pi r_{1}^{3}}{3V_{\text{s}}}$, where *x*_sol_ is the solvent concentration within the PHEMA cores. HEMA monomer is immiscible with PAO at 20°C, so this solvent is clearly a very poor solvent for the PHEMA chains. Accordingly, *x*_sol_ was set to zero for SAXS analysis. The amplitude of the core self-term is expressed as:

$A_{\text{s}}(q,r_{1})=\Phi(qr_{1})\exp\left(-\frac{q^{2}\sigma^{2}}{2}\right)$ (S17)

where $\Phi(qr_{1})=\frac{3[\sin(qr_{1})-qr_{1}\cos(qr_{1})]}{(qr_{1})^{3}}$ is the form factor amplitude of a sphere. In this case, the form factor of the micelle core in eq. S15 is expressed as:

$F_{\text{s}}(q,r_{1})=A_{\text{s}}^{2}(q,r_{1})$ (S18)

The exponent term in eq. S17 represents a sigmoidal interface between the blocks with a width *σ* with a decaying scattering length density at the core surface. This *σ* value was fixed at 0.22 nm during SAXS data fitting. The self-correlation term for the corona block is given by the Debye function:

$F_{\text{c}}(qR_{\text{g}})=\frac{2[\exp(-q^{2}R_{\text{g}}^{2})-1+q^{2}R_{\text{g}}^{2}]}{q^{4}R_{\text{g}}^{4}}$ (S19)

The amplitude of the corona chain form factor is obtained from a normalized Fourier transform of the radial density distribution function of the corona chains:

$A_{\text{c}}(q,r_{1})=\frac{\int_{r_{1}}^{r_{1}+2s} \mu_{\text{c}}(r)\frac{\sin(qr)}{qr}r^{2}dr}{\int_{r_{1}}^{r_{1}+2s} \mu_{\text{c}}(r)r^{2}dr}\exp\left(-\frac{q^{2}\sigma^{2}}{2}\right)$ (S20) The radial profile, *μ*_c_(*r*), can be expressed by a linear combination of two cubic B-splines, with two fitting parameters *s* and *a* corresponding to the width of the corona electron density profile and the function weight coefficient, respectively. It was constrained when analyzing the SAXS patterns such that $s=2R_{\text{g}}$. This information can be found elsewhere,^1,2^ along with the approximate integrated form of eq. S20. Finally, *N* in eq. S14 is expressed as:

(S21)

where *ϕ* is the total volume fraction of diblock copolymer and $V(r_{1})$ is the total volume occupied by a spherical micelle excluding the surrounding solvent. For spherical micelles, the latter parameter is expressed in terms of the volume occupied by each block (i.e., *V*_s_ and *V*_c_): $V(r_{1})=(V_{\text{s}}+V_{\text{c}})N_{\text{agg}}(r_{1})$.

To analyze the scattering pattern recorded for the nanoparticles swollen with an aqueous solution of 0.3 M HCl, the spherical micelle form factor (eq. S15) was modified by replacing$N_{\text{agg}}(r_{1})\beta_{\text{s}}$ with a$\frac{4\pi r_{1}^{3}}{3}(\xi_{\text{s+HCl}}-\xi_{\text{sol}})$ term, and $\xi_{\text{s+HCl}}=(1-x_{\text{HCl}})\xi_{\text{s}}+x_{\text{HCl}}\xi_{\text{HCl}}$, where $x_{\text{HCl}}$ is the volume fraction of the aqueous 0.3 M HCl solution present in the swollen micelle core (calculated using $x_{\text{HCl}}=1-\overline{N_{\text{agg}}}\frac{3V_{\text{s}}}{4\pi R_{\text{s}}^{3}}$, where $\overline{N_{\text{agg}}}$ is the mean aggregation number of the micelles as calculated using the mean core radius of the original non-swollen micelles determined by SAXS) and $\xi_{\text{HCl}}$ is the SLD of the aqueous 0.3 M HCl solution (9.43 × 10^10^ cm^-2^) calculated using the solution composition and its density (1.002 g cm^-3^) as measured by solution densitometry. In this case, the nanoparticle volume in eq. S21 is expressed as $V(r_{1})=\frac{4\pi r_{1}^{3}}{3}+V_{\text{c}}N_{\text{agg}}(r_{1})$ where it is assumed that no PAO solvent molecules penetrate the polar micelle cores (*x*_sol_ = 0) and $N_{\text{agg}}(r_{1})=(1-x_{\text{HCl}})\frac{4\pi r_{1}^{3}}{3V_{\text{s}}}$. This analysis assumes that the mean micelle aggregation number does not change during acid swelling. This is a reasonable assumption since the PHEMA chains are almost certainly lightly crosslinked owing to the presence of a low level of dimethacrylate impurity in the HEMA monomer.^[2]^

Scattering patterns recorded for the silica-loaded or titania-loaded nanoparticle dispersions were analyzed using the same spherical micelle form factor (eq. S15). However, the presence of the inorganic component within the micelle cores should be accounted for in this model. According to Pedersen et al., additional scattering from embedded blobs can be described by incorporating the scattering amplitude and form factor for a Gaussian chain into the scattering equation.^[11]^ Following the established conventions for formulating scattering intensity expressions for copolymer micelle models,^[6,12]^ and bearing in mind a recent scattering study of composite casein micelles,^[13]^ the scattering amplitude and form factor for such inorganic oxide-loaded micelle cores can be written as:

$A_{\text{s}}(q,r_{1})=[(\xi_{\text{s}}-\xi_{\text{sol}})\Phi^{2}(qr_{1})\frac{4\pi r_{1}^{3}}{3}+(\xi_{\text{in}}-\xi_{\text{s}})n_{\text{in}}V_{\text{in}}\psi(q,R_{\text{gin}})]/\left(\frac{4\pi r_{1}^{3}}{3}\right)$ (S22)

$\begin{matrix} F_{\text{s}}(q,r_{1})=[(\xi_{\text{s}}-\xi_{\text{sol}})^{2}\Phi^{2}(qr_{1})\left(\frac{4\pi r_{1}^{3}}{3}\right)^{2}+ \\ +2(\xi_{\text{in}}-\xi_{\text{s}})(\xi_{\text{s}}-\xi_{\text{sol}})n_{\text{in}}V_{\text{in}}\frac{4\pi r_{1}^{3}}{3}\psi(qR_{\text{gin}})\Phi^{2}(qr_{1})+ \\ +(\xi_{\text{in}}-\xi_{\text{s}})^{2}n_{\text{in}}(n_{\text{in}}-1)V_{\text{in}}^{2}\psi^{2}(qR_{\text{gin}})\Phi^{2}(qr_{1})+ \\ +(\xi_{\text{in}}-\xi_{\text{s}})^{2}n_{\text{in}}V_{\text{in}}^{2}F_{\text{c}}(qR_{\text{gin}})]/\left(\frac{4\pi r_{1}^{3}}{3}\right)^{2} \end{matrix}$ (S23)

where *R*_gin_ is the radius of gyration of the inorganic oxide component, $\psi(qR_{\text{gin}})=\frac{1-\exp(q^{2}R_{\text{gin}}^{2})}{q^{2}R_{\text{gin}}^{2}}$ is the scattering amplitude of its Gaussian chain,^[14]^ $V_{\text{in}}=\frac{4\pi R_{\text{gin}}^{3}}{3}$ is the approximate volume of a single blob, $n_{\text{in}}=\frac{1}{V_{\text{in}}}\left(\frac{4\pi r_{1}^{3}}{3}(1-x_{\text{sol}})-N_{\text{agg}}(r_{1})V_{\text{c}}\right)$ is the mean number of blobs per micelle core and *ξ*_in_ is X-ray scattering length density of the blob calculated from their assumed chemical composition (i.e., SiO_2_ or TiO_2_) and mass and X-ray scattering densities (*ρ*_SiO2_ = 1.6 g cm^-3^ and *ξ*_SiO2_ = 13.56 × 10^10^ cm^-2^; *ρ*_TiO2_ = 2.2 g cm^-3^ and *ξ*_TiO2_ = 17.76 × 10^10^ cm^-2^). The mass densities were estimated from solution densitometry measurements conducted on a series of aqueous suspensions of silica and titania prepared at various concentrations. Like the acid-swollen nanoparticles, the volume of such silica-loaded (or titania-loaded) nanoparticles for eq. S21 is expressed as $V(r_{1})=\frac{4\pi r_{1}^{3}}{3}+V_{\text{c}}N_{\text{agg}}(r_{1})$ assuming that no PAO molecules are located within the nanoparticle cores (*x*_sol_ = 0) and $N_{\text{agg}}(r_{1})=(1-x_{\text{in}})\frac{4\pi r_{1}^{3}}{3V_{\text{s}}}$, where the volume fraction of the inorganic oxide component, *x*_in_, within the nanoparticle cores is given by $x_{\text{in}}=1-\overline{N_{\text{agg}}}\frac{3V_{\text{s}}}{4\pi R_{\text{s}}^{3}}$. Again, this analysis assumes that the mean micelle aggregation number remains unchanged after silica (or titania) loading.

**Table S3.** Summary of structural parameters calculated from SAXS analysis of the PLMA_194_-PHEMA_x_ nanoparticles. *R*_s_ is the mean spherical micelle core radius and *σ_R_*_s_ is its standard deviation, *s* is the corona thickness (i.e., the width of the corona electron density profile), *N*_agg_(*R*_s_) is the mean aggregation number of the micelles and *S*_block_(*R*_s_) is the mean area occupied by each coronal PLMA_194_ chain at the nanoparticle surface as calculated using $S_{\text{block}}(R_{\text{s}})=\pi R_{\text{s}}^{2}/N_{\text{agg}}(R_{\text{s}})$

| **Sample ID** | ***R*_s_, Å** | ***σ_R_*_s_, Å** | ***s*, Å** | ***N*_agg_(*R*_s_)** | ***S*_block_(*R*_s_), Å^2^** |
| --- | --- | --- | --- | --- | --- |
| PLMA_194_-PHEMA_980_ | 293 | 30 | 52 | 637 | 423 (20.6×20.6) |
| PLMA_194_-PHEMA_1500_ | 321 | 33 | 45 | 547 | 592 (24.3×24.3) |
| PLMA_194_-PHEMA_2000_ | 365 | 35 | 40 | 603 | 694 (26.3×26.3) |
| PLMA_194_-PHEMA_2910_ | 406 | 32 | 30 | 571 | 907 (30.1×30.1) |
| PLMA_194_-PHEMA_2000_ + HCl | 399 | 27 | 40* | 603* | 829 (28.8×28.8)* |
| PLMA_194_-PHEMA_2000_ + SiO_2_ | 426 | 29 | 40* | 603* | 945 (30.7×30.7)* |
| PLMA_194_-PHEMA_2000_ + TiO_2_^$^ | 365 | 41 | - | - | - |

* These parameters were calculated from SAXS analysis of the original PLMA_194_-PHEMA_2000_ nanoparticles and were fixed when fitting SAXS patterns.

$ SAXS data obtained using a standard core-shell model (see main manuscript for details).

**References**

[1]	M. J. Derry, L. A. Fielding, S. P. Armes, “Industrially-relevant polymerization-induced self-assembly formulations in non-polar solvents: RAFT dispersion polymerization of benzyl methacrylate” *Polym. Chem.* **2015**, *6*, 3054–3062.

[2]	P. Chohan, C. György, O. O. Mykhaylyk, G. M. Prentice, S. V. Filip, M. J. Payne, G. Manna, S. P. Armes, “RAFT Dispersion Polymerization of 2-Hydroxyethyl Methacrylate in Non-polar Media” *Macromolecules* **2024**, *57*, 11738–11752.

[3]	J. S. Trent, “Ruthenium Tetraoxide Staining of Polymers: New Preparative Methods for Electron Microscopy” *Macromolecules* **1984**, *17*, 2930–2931.

[4]	M. Basham, J. Filik, M. T. Wharmby, P. C. Y. Chang, B. El Kassaby, M. Gerring, J. Aishima, K. Levik, B. C. A. Pulford, I. Sikharulidze, D. Sneddon, M. Webber, S. S. Dhesi, F. Maccherozzi, O. Svensson, S. Brockhauser, G. Náray, A. W. Ashton, “Data Analysis WorkbeNch (DAWN)” *J. Synchrotron Radiat.* **2015**, *22*, 853–858.

[5]	J. Ilavsky, Peter. R. Jemian, “Irena: tool suite for modeling and analysis of small-angle scattering” *J. Appl. Crystallogr.* **2009**, *42*, 347–353.

[6]	J. S. Pedersen, “Form factors of block copolymer micelles with spherical, ellipsoidal and cylindrical cores” *J. Appl. Crystallogr.* **2000**, *33*, 637–640.

[7]	R.-J. Roe, *Methods of X-ray and Neutron Scattering in Polymer Science*, Oxford University Press, New York, **2000**.

[8]	E. J. Quane, N. Elders, A. S. Newman, S. van Mourik, N. S. J. Williams, K. J. van den Berg, A. J. Ryan, O. O. Mykhaylyk, “Synthesis, Morphology, and Particle Size Control of Acidic Aqueous Polyurethane Dispersions” *Macromolecules* **2024**, *57*, 10623–10634.

[9]	J. S. Pedersen, M. C. Gerstenberg, “The structure of P85 Pluronic block copolymer micelles determined by small-angle neutron scattering” *Colloids Surf. A Physicochem. Eng. Asp.* **2003**, *213*, 175–187.

[10]	J. Skov Pedersen, C. Svaneborg, K. Almdal, I. W. Hamley, R. N. Young, “A Small-Angle Neutron and X-ray Contrast Variation Scattering Study of the Structure of Block Copolymer Micelles: Corona Shape and Excluded Volume Interactions” *Macromolecules* **2003**, *36*, 416–433.

[11]	J. S. Pedersen, I. W. Hamley, C. Y. Ryu, T. P. Lodge, “Contrast Variation Small-Angle Neutron Scattering Study of the Structure of Block Copolymer Micelles in a Slightly Selective Solvent at Semidilute Concentrations” *Macromolecules* **2000**, *33*, 542–550.

[12]	J. S. Pedersen, “Structure factors effects in small-angle scattering from block copolymer micelles and star polymers” *J. Chem. Phys.* **2001**, *114*, 2839–2846.

[13]	J. S. Pedersen, T. L. Møller, N. Raak, M. Corredig, “A model on an absolute scale for the small-angle X-ray scattering from bovine casein micelles” *Soft Matter* **2022**, *18*, 8613–8625.

[14]	B. Hammouda, “Structure factor for starburst dendrimers” *J. Polym. Sci. B Polym. Phys.* **1992**, *30*, 1387–1390.
